# A functional genomics screen identifying blood cell development genes in *Drosophila* by undergraduates participating in a course-based research experience

**DOI:** 10.1093/g3journal/jkaa028

**Published:** 2021-01-30

**Authors:** Cory J Evans, John M Olson, Bama Charan Mondal, Pratyush Kandimalla, Ariano Abbasi, Mai M Abdusamad, Osvaldo Acosta, Julia A Ainsworth, Haris M Akram, Ralph B Albert, Elitzander Alegria-Leal, Kai Y Alexander, Angelica C Ayala, Nataliya S Balashova, Rebecca M Barber, Harmanjit Bassi, Sean P Bennion, Miriam Beyder, Kush V Bhatt, Chinmay Bhoot, Aaron W Bradshaw, Tierney G Brannigan, Boyu Cao, Yancey Y Cashell, Timothy Chai, Alex W Chan, Carissa Chan, Inho Chang, Jonathan Chang, Michael T Chang, Patrick W Chang, Stephen Chang, Neel Chari, Alexander J Chassiakos, Iris E Chen, Vivian K Chen, Zheying Chen, Marsha R Cheng, Mimi Chiang, Vivian Chiu, Sharon Choi, Jun Ho Chung, Liset Contreras, Edgar Corona, Courtney J Cruz, Renae L Cruz, Jefferson M Dang, Suhas P Dasari, Justin R O De La Fuente, Oscar M A Del Rio, Emily R Dennis, Petros S Dertsakyan, Ipsita Dey, Rachel S Distler, Zhiqiao Dong, Leah C Dorman, Mark A Douglass, Allysen B Ehresman, Ivy H Fu, Andrea Fua, Sean M Full, Arash Ghaffari-Rafi, Asmar Abdul Ghani, Bosco Giap, Sonia Gill, Zafar S Gill, Nicholas J Gills, Sindhuja Godavarthi, Talin Golnazarian, Raghav Goyal, Ricardo Gray, Alexander M Grunfeld, Kelly M Gu, Natalia C Gutierrez, An N Ha, Iman Hamid, Ashley Hanson, Celesti Hao, Chongbin He, Mengshi He, Joshua P Hedtke, Ysrael K Hernandez, Hnin Hlaing, Faith A Hobby, Karen Hoi, Ashley C Hope, Sahra M Hosseinian, Alice Hsu, Jennifer Hsueh, Eileen Hu, Spencer S Hu, Stephanie Huang, Wilson Huang, Melanie Huynh, Carmen Javier, Na Eun Jeon, Sunjong Ji, Jasmin Johal, Amala John, Lauren Johnson, Saurin Kadakia, Namrata Kakade, Sarah Kamel, Ravinder Kaur, Jagteshwar S Khatra, Jeffrey A Kho, Caleb Kim, Emily Jin-Kyung Kim, Hee Jong Kim, Hyun Wook Kim, Jin Hee Kim, Seong Ah Kim, Woo Kyeom Kim, Brian Kit, Cindy La, Jonathan Lai, Vivian Lam, Nguyen Khoi Le, Chi Ju Lee, Dana Lee, Dong Yeon Lee, James Lee, Jason Lee, Jessica Lee, Ju-Yeon Lee, Sharon Lee, Terrence C Lee, Victoria Lee, Amber J Li, Jialing Li, Alexandra M Libro, Irvin C Lien, Mia Lim, Jeffrey M Lin, Connie Y Liu, Steven C Liu, Irene Louie, Shijia W Lu, William Y Luo, Tiffany Luu, Josef T Madrigal, Yishan Mai, Darron I Miya, Mina Mohammadi, Sayonika Mohanta, Tebogo Mokwena, Tonatiuh Montoya, Dallas L Mould, Mark R Murata, Janani Muthaiya, Seethim Naicker, Mallory R Neebe, Amy Ngo, Duy Q Ngo, Jamie A Ngo, Anh T Nguyen, Huy C X Nguyen, Rina H Nguyen, Thao T T Nguyen, Vincent T Nguyen, Kevin Nishida, Seo-Kyung Oh, Kristen M Omi, Mary C Onglatco, Guadalupe Ortega Almazan, Jahzeel Paguntalan, Maharshi Panchal, Stephanie Pang, Harin B Parikh, Purvi D Patel, Trisha H Patel, Julia E Petersen, Steven Pham, Tien M Phan-Everson, Megha Pokhriyal, Davis W Popovich, Adam T Quaal, Karl Querubin, Anabel Resendiz, Nadezhda Riabkova, Fred Rong, Sarah Salarkia, Nateli Sama, Elaine Sang, David A Sanville, Emily R Schoen, Zhouyang Shen, Ken Siangchin, Gabrielle Sibal, Garuem Sin, Jasmine Sjarif, Christopher J Smith, Annisa N Soeboer, Cristian Sosa, Derek Spitters, Bryan Stender, Chloe C Su, Jenny Summapund, Beatrice J Sun, Christine Sutanto, Jaime S Tan, Nguon L Tan, Parich Tangmatitam, Cindy K Trac, Conny Tran, Daniel Tran, Duy Tran, Vina Tran, Patrick A Truong, Brandon L Tsai, Pei-Hua Tsai, C Kimberly Tsui, Jackson K Uriu, Sanan Venkatesh, Maique Vo, Nhat-Thi Vo, Phuong Vo, Timothy C Voros, Yuan Wan, Eric Wang, Jeffrey Wang, Michael K Wang, Yuxuan Wang, Siman Wei, Matthew N Wilson, Daniel Wong, Elliott Wu, Hanning Xing, Jason P Xu, Sahar Yaftaly, Kimberly Yan, Evan Yang, Rebecca Yang, Tony Yao, Patricia Yeo, Vivian Yip, Puja Yogi, Gloria Chin Young, Maggie M Yung, Alexander Zai, Christine Zhang, Xiao X Zhang, Zijun Zhao, Raymond Zhou, Ziqi Zhou, Mona Abutouk, Brian Aguirre, Chon Ao, Alexis Baranoff, Angad Beniwal, Zijie Cai, Ryan Chan, Kenneth Chang Chien, Umar Chaudhary, Patrick Chin, Praptee Chowdhury, Jamlah Dalie, Eric Y Du, Alec Estrada, Erwin Feng, Monica Ghaly, Rose Graf, Eduardo Hernandez, Kevin Herrera, Vivien W Ho, Kaitlyn Honeychurch, Yurianna Hou, Jo M Huang, Momoko Ishii, Nicholas James, Gah-Eun Jang, Daphne Jin, Jesse Juarez, Ayse Elif Kesaf, Sat Kartar Khalsa, Hannah Kim, Jenna Kovsky, Chak Lon Kuang, Shraddha Kumar, Gloria Lam, Ceejay Lee, Grace Lee, Li Li, Joshua Lin, Josephine Liu, Janice Ly, Austin Ma, Hannah Markovic, Cristian Medina, Jonelle Mungcal, Bilguudei Naranbaatar, Kayla Patel, Lauren Petersen, Amanda Phan, Malcolm Phung, Nadiyah Priasti, Nancy Ruano, Tanveer Salim, Kristen Schnell, Paras Shah, Jinhua Shen, Nathan Stutzman, Alisa Sukhina, Rayna Tian, Andrea Vega-Loza, Joyce Wang, Jun Wang, Rina Watanabe, Brandon Wei, Lillian Xie, Jessica Ye, Jeffrey Zhao, Jill Zimmerman, Colton Bracken, Jason Capili, Andrew Char, Michel Chen, Pingdi Huang, Sena Ji, Emily Kim, Kenneth Kim, Julie Ko, Sean Louise G Laput, Sam Law, Sang Kuk Lee, Olivia Lee, David Lim, Eric Lin, Kyle Marik, Josh Mytych, Andie O'Laughlin, Jensen Pak, Claire Park, Ruth Ryu, Ashwin Shinde, Manny Sosa, Nick Waite, Mane Williams, Richard Wong, Jocelyn Woo, Jonathan Woo, Vishaal Yepuri, Dorothy Yim, Dan Huynh, Dinali Wijiewarnasurya, Casey Shapiro, Marc Levis-Fitzgerald, Leslie Jaworski, David Lopatto, Ira E Clark, Tracy Johnson, Utpal Banerjee

**Affiliations:** 1 Department of Molecular, Cell, and Developmental Biology, University of California, Los Angeles, Los Angeles, CA 90095, USA; 2 Biomedical Research Minor, University of California, Los Angeles, Los Angeles, CA 90095, USA; 3 Center for the Advancement of Teaching, University of California, Los Angeles, Los Angeles, CA 90095, USA; 4 Department of Psychology, Grinnell College, Grinnell, IA 50112, USA; 5 Department of Biological Chemistry, University of California, Los Angeles, Los Angeles, CA 90095, USA; 6 UCLA Broad Stem Cell Research Center, University of California, Los Angeles, Los Angeles, CA 90095, USA

**Keywords:** hematopoiesis, blood, RNAi, education, CURE

## Abstract

Undergraduate students participating in the UCLA Undergraduate Research Consortium for Functional Genomics (URCFG) have conducted a two-phased screen using RNA interference (RNAi) in combination with fluorescent reporter proteins to identify genes important for hematopoiesis in *Drosophila*. This screen disrupted the function of approximately 3500 genes and identified 137 candidate genes for which loss of function leads to observable changes in the hematopoietic development. Targeting RNAi to maturing, progenitor, and regulatory cell types identified key subsets that either limit or promote blood cell maturation. Bioinformatic analysis reveals gene enrichment in several previously uncharacterized areas, including RNA processing and export and vesicular trafficking. Lastly, the participation of students in this course-based undergraduate research experience (CURE) correlated with increased learning gains across several areas, as well as increased STEM retention, indicating that authentic, student-driven research in the form of a CURE represents an impactful and enriching pedagogical approach.

## Introduction

The Undergraduate Research Consortium for Functional Genomics (URCFG) was established at UCLA in 2003 as an entity representing the collaborative research effort of undergraduates, typically first- and second-year students, participating in a discovery-based laboratory course called *Biomedical Research 10H* (formerly *Life Sciences 10H*). Since that time, the URCFG has conducted several large-scale genetic research projects that have yielded publishable data and research resources (Chen *et al.* 2005; Liao *et al.* 2006; Call *et al.* 2007; Evans *et al.* 2009; Olson *et al.* 2019).

The current URCFG research project centers on the discovery of new genes controlling hematopoiesis (blood formation) in the fruit fly, *Drosophila melanogaster*. Over the last two decades, the fly has become an increasingly popular model for investigating the molecular mechanisms regulating blood cell specification, development, and function (Evans *et al.* 2003; Gold and Brückner 2014; Letourneau *et al.* 2016; Banerjee *et al.* 2019). This is due in large part to the established strength of *Drosophila* genetics and many developmental and functional parallels between human and fly blood systems. From a relative perspective, the development of the human blood system is extremely well understood, owing to a long history of observational and functional studies *ex vivo*, the development of blood and bone marrow transplant technologies in medicine, and the creation and analyses of a variety of highly relevant models such as the mouse and, more recently, zebrafish. Nevertheless, the human blood system is highly complex, and much is still to be learned about the genes that control development and, when disrupted, cause disease.

In both flies and humans, mature blood cell types are derived from progenitor cells through highly regulated differentiation. In humans, multipotent hematopoietic stem cells (HSCs) give rise to blood progenitors that belong to either myeloid or lymphoid lineage, which further differentiate into a variety of mature forms (Orkin and Zon 2008). Likewise, multipotent progenitor cells give rise to the mature blood cell types in *Drosophila* (Jung *et al.* 2005), although it is still unclear whether true blood stem cells are present in the fly. The origin of *Drosophila* blood cells (also called hemocytes) occurs in two separate specification events that differ in space and time. The first wave of hematopoiesis occurs in the embryonic head mesoderm and creates blood cells that quickly mature and migrate throughout the developing embryo, eventually becoming the circulating blood cells of the larva. A subset of these cells, many of which appear to retain progenitor characteristics, become sessile, attaching to the lateral body wall around the chordotonal organs and to various internal organs (Márkus *et al.* 2009; Makhijani *et al.* 2011; Leitão and Sucena 2015). The second is the independent wave of blood cell specification, which begins slightly later in the embryonic cardiogenic mesoderm and contributes early blood progenitors that collectively form a specialized, multi-lobed organ called the lymph gland. During the larval stages, the lymph gland grows in size as these blood progenitors proliferate, and in the mid-second instar, a subset of these cells begin to differentiate (Jung *et al.* 2005). By the late third instar, the lymph gland primary lobes (the largest and the most anterior) contain organized, spatially restricted populations of mature and progenitor blood cells that occupy the Cortical Zone (CZ) and Medullary Zone (MZ), respectively (Jung *et al.* 2005). Additionally, a small group of dedicated regulatory cells, called the Posterior Signaling Center (PSC), is located at the posterior end of the primary lobes and influences progenitor cell maintenance and differentiation (Lebestky *et al.* 2003; Sinenko and Mathey-Prevot 2004; Jung *et al.* 2005; Krzemień *et al.* 2007; Mandal *et al.* 2007; Tokusumi *et al.* 2010, 2012, 2015).

*Drosophila* has three defined terminally differentiated blood cell types called plasmatocytes, crystal cells, and lamellocytes (Evans *et al.* 2014; Olson *et al.* 2019). Plasmatocytes are professional phagocytes, similar to human macrophages and neutrophils, and are by far the most prevalent blood cell type (∼95%) produced. Crystal cells make up most of the remainder and have roles in blood coagulation, sclerotization, and melanization, reminiscent of the role of megakaryocytes and derivative platelets in clotting. Lamellocytes are large, flat cells that are rare under normal developmental conditions, but can be induced to develop upon immune challenge. In the wild, fly larvae are the targets of parasitoid wasps that inject their embryos into the body cavity. In response, *Drosophila* larvae produce lamellocytes that, in conjunction with plasmatocytes and crystal cells, isolate and kill the wasp embryo through encapsulation, much like granuloma formation by specialized macrophages in humans (Rizki and Rizki 1992; Cronan *et al.* 2016). Thus, *Drosophila* blood cells exhibit key functional similarities to cells of the human myeloid lineage (Bidla *et al.* 2007; Buchon *et al.* 2014; Gold and Brückner 2014, 2015).

With regard to the genetic control of hematopoietic development, numerous studies have highlighted the conserved function of important signaling systems and gene expression regulators between *Drosophila* and humans (Evans *et al.* 2003, 2014; Banerjee *et al.* 2019). For example, mesodermal formation of the *Drosophila* lymph gland and the mammalian aorta-gonadal-mesonephros (AGM) region, from which early blood cells are derived, both require FGF, BMP, and Wnt signaling (Mandal *et al.* 2004). Additionally, blood cell specification and lineage commitment in both flies and mammals require the function of GATA and Runx family transcriptional regulators (Daga *et al.* 1996; Rehorn *et al.* 1996; Lebestky *et al.* 2000; Han and Olson 2005). Other conserved transcription factors, including HOX, FOG, and EBF homologs (Fossett *et al.* 2001; Crozatier *et al.* 2004; Mandal *et al.* 2007), have also been shown to share regulatory roles. The activity of such factors are themselves regulated by an assortment of signaling pathways, such as the Pvr, FGF, and EGF receptor tyrosine kinase (Brückner *et al.* 2004; Jung *et al.* 2005; Mondal *et al.* 2011; Sinenko *et al.* 2012; Dragojlovic-Munther and Martinez-Agosto 2013), JAK/STAT (Harrison *et al.* 1995; Luo *et al.* 2002), Notch (Duvic *et al.* 2002; Lebestky *et al.* 2003), Wingless (Sinenko *et al.* 2009), and Hedgehog pathways (Mandal *et al.* 2007), which are also conserved.

Though our understanding of the genetic control of hematopoietic development in *Drosophila* continues to grow, what is known is extremely limited from a genomic perspective. Most of the hematopoietic genes that have been identified to date stem from trial-and-error analysis of important genes known from other contexts, and a small number of forward genetic screens that produced discernible hematopoietic phenotypes. Sequencing of the fly genome has identified almost 14,000 protein coding genes, but which subset of the genome regulates hematopoietic development is largely unknown. Thus, the URCFG initiated a functional genomics project, in which reverse genetic analysis was used to link *Drosophila* genes to hematopoiesis. Moreover, by engaging in authentic research experiences, students show compelling learning outcomes, even when compared with students in traditional laboratory courses or summer laboratory apprenticeships.

## Materials and methods

### GAL4 driver lines

For the primary screen (expression throughout the hematopoietic system), the *HHLT-GAL4 UAS-GFP* line *{Hand-GAL4 Hml^Δ^-GAL4 UAS-FLP.JD1 UAS-2XEGFP; P[GAL4-Act5C(FRT.CD2).P]S}* Chr. (2; 3) was used as previously described (Mondal *et al.* 2014). For the secondary screen (expression in lymph gland sub-populations), lines containing *Antp-GAL4* (Mandal *et al.* 2007), or *dome-GAL4* (Jung *et al.* 2005; Yoon *et al.* 2017), or *Hml^Δ^-GAL4* (Sinenko and Mathey-Prevot 2004; Jung *et al.* 2005) were used to target RNAi to PSC, progenitor, and differentiating/mature cells, respectively. The *Hml^Δ^-DsRed* (Makhijani *et al.* 2011) reporter was used to identify differentiating and mature blood cells. Specific genotypes were as follows: *Hml^Δ^-DsRed/CyO*; *Antp-GAL4 UAS-GFP/TM6B Tb*, *elav-GAL80; Hml^Δ^-DsRed/*; *Antp-GAL4 UAS-GFP/SM6a-TM6B Tb*, *dome-GAL4^PG14^ UAS-GFP/FM7i*, *Hml^Δ^-DsRed/CyO*, *elav-GAL80*; *Hml^Δ^-DsRed/*; *dome^MESO^-GAL4/SM6a-TM6B Tb*, and *Hml^Δ^-DsRed Hml^Δ^-GAL4/CyO*. For controls, *GAL4* drivers were crossed with *white^1118^* (BDSC 5905).

### RNAi lines

Transgenic RNAi lines for screening were obtained from the Vienna *Drosophila* RNAi Center (VDRC, Vienna, Austria; GD and KK collection), the National Institute of Genetics (Kyoto, Japan; NIG-R lines), and the Bloomington *Drosophila* Stock Center (BDSC, Bloomington, Indiana; TRiP lines). Acquired RNAi lines were randomly assigned to students participating in the primary screen and the secondary screen, and each RNAi line was assigned to a minimum of two students. Each RNAi line was continually screened until two complete data sets (see below) were acquired. For target gene validation, the BDSC was searched for alternate RNAi lines targeting 24 candidate genes identified by *Hml^Δ^-GAL4* in our secondary screen (those causing strong increases in *Hml^Δ^-DsRed* fluorescence); 14 alternative RNAi lines were available, obtained, and screened (Supplementary Figure S1).

### Crossing conditions

Virgin *GAL4* females were crossed to males from individual *UAS-hpRNA* lines or to males from *w^1118^* (BDSC 5905) as a control. Crosses to *HHLT-GAL4* and *Hml^Δ^-GAL4* were reared at 29°C to maximize RNAi-based phenotypes. Crosses to *Antp-GAL4* and *dome^PG14^-GAL4* were placed directly at 29°C or reared for one day at 18°C before shifting to 29°C. Crosses to *Antp-GAL4* and *dome^MESO^-GAL4* with *elav-GAL80* were reared at room temperature for one day before shifting to 29°C.

### Processing and imaging of larvae

Wandering third-instar larvae (non-Tb) were collected, washed with water, and placed into glass spot well plates (Fisher) on ice to minimize movement. Depending upon balancer chromosomes present in the parental *GAL4* driver line, larvae were sometimes prescreened for the presence of GFP and DsRed expression. Four immobile larvae were aligned dorsal side up along the anterior/posterior axis on the bottom (flat surface) of a glass spot well plate that was chilled on ice. Larvae were then imaged for GFP or DsRed fluorescence using a Zeiss Stemi SV11 fluorescence stereo dissection microscope (1.0× objective lens, 0.8× magnification) equipped with an AxioCam MRm camera, controlled by Zeiss AxioVision imaging software. Imaging 12 larvae (three sets of four larvae) for each cross was considered as a complete dataset.

### Phenotype screening in whole animals

Reporter gene expression (fluorescence) in progeny larvae activating RNAi within the hematopoietic system was compared with that of progeny larvae in which RNAi was absent (from control crosses). For the primary (*HHLT-GAL4 UAS-GFP*) screen, students noted changes to fluorescence associated with the lymph gland region, including the posterior pericardial cells, and the circulating blood cell population. Changes noted were varying levels of increased or decreased fluorescence for lymph glands (including missing or partially missing), whether pericardial cells were absent, increased or decreased circulating cell density (including clumps and melanotic tumors). For the secondary screen with *Hml^Δ^-DsRed* as a marker, students noted changes to fluorescence associated with the lymph gland region and the circulating blood cell population. Changes noted were varying levels of increased or decreased fluorescence for lymph glands (including missing or partially missing) and increased or decreased circulating cell density (including clumps and melanotic tumors). RNAi phenotypes were scored by two or more students in both the primary and the secondary screens, with “hits” being selected by causing reproducible phenotype scores at each stage. Because circulating cell phenotypes varied in several ways, scoring was more subjective. Thus, RNAi line reproducibly causing circulating cell phenotypes were consolidated into a single group that cause any relative change (Supplementary Table S3).

### Bioinformatic analysis

For RNAi lines causing a developmental phenotype, associated target genes were identified through their respective stock center databases. Gene information and protein sequences were retrieved from FlyBase (Attrill *et al.* 2016). Potential human homologs were identified using the Basic Local Alignment Search Tool (BLAST; National Center for Biotechnology Information) featuring the protein: protein BLAST (blastp) algorithm. Functional annotation of genes was performed using the STRING protein–protein interaction database (v11.0; Szklarczyk *et al.* 2019), which also includes the Kyoto Encyclopedia of Genes and Genomes (KEGG) Pathway database (Kanehisa and Goto 2000) and Reactome database (Fabregat *et al.* 2018) as analysis tools.

### Assessment of learning gains

Learning gains were assessed using the Survey of Undergraduate Research Experiences (SURE) II (Lopatto 2004), which offers both the Classroom Undergraduate Research Experiences (CURE) survey and the Summer Undergraduate Research Experience (SURE) survey. The CURE and the SURE surveys include identical items that permit comparisons; URCFG students and the “All students” group took the CURE survey, while the “All summer research students” group took the SURE survey. A total of 308 UCLA undergraduates participating in this URCFG RNAi CURE project identified as follows: 53.9% female (*n* = 166), 46.1% male (*n* = 142); of 294 respondents, 10.1% were URM (*n* = 31), where URM includes American Indian/Alaskan Native, Black/African American, or Hispanic/Latinx; student make-up by year: first-year, 33.1% (*n* = 102), second-year, 41.6% (*n* = 128), third-year, 20.8% (*n* = 64), and fourth-year, 4.5% (*n* = 14). SURE II survey participants (January 2015 through May 2018) identified as follows: of 17,810 respondents, 64.6% were female (*n* = 11,512), 35.4% were male (*n* = 6298); of 17,638 respondents, 17.8% were URM (*n* = 3142); of 17,328 respondents, student make-up by year: first-year, 35.2% (*n* = 6103), second-year, 26.2% (*n* = 4547), third-year, 20.2% (*n* = 3496), and fourth-year, 18.4% (*n* = 3182). UCLA student demographic data were obtained under UCLA IRB#16-001388.

### Reagent and data availability

*GAL4* driver lines are available upon request from the Biomedical Research Minor and Banerjee laboratory (UCLA). RNAi lines are available from the Bloomington *Drosophila* Stock Center (Bloomington, IN), NIG-FLY, National Institute of Genetics (Japan), and the Vienna *Drosophila* Resource Center (Austria). Supplementary Figure S1 shows phenotypic validation data for a subset of RNAi lines. Supplementary Table S1 contains a list of additional RNAi lines added to the collection identified by the primary phase of the genetic screen. Supplementary Table S2 lists the duplicate screening completion rate for each *GAL4* driver/RNAi line combination. Supplementary Table S3 lists candidate genes regulating circulating blood cells. Supplementary Table S4 list Gene Ontology (GO) terms enriched among genes identified in the primary phase screen with *HHLT-GAL4*. Supplementary Table S5 lists all enriched Reactome groups among genes identified in the primary phase screen with *HHLT-GAL4*. Supplementary Figure S1 is in TIF format. All Supplementary Tables are in Microsoft Excel (.xlsx) format and have been uploaded to figshare: https://doi.org/10.25387/g3.13166891.

## Results

### Identification of new hematopoietic genes

**Figure 1:**
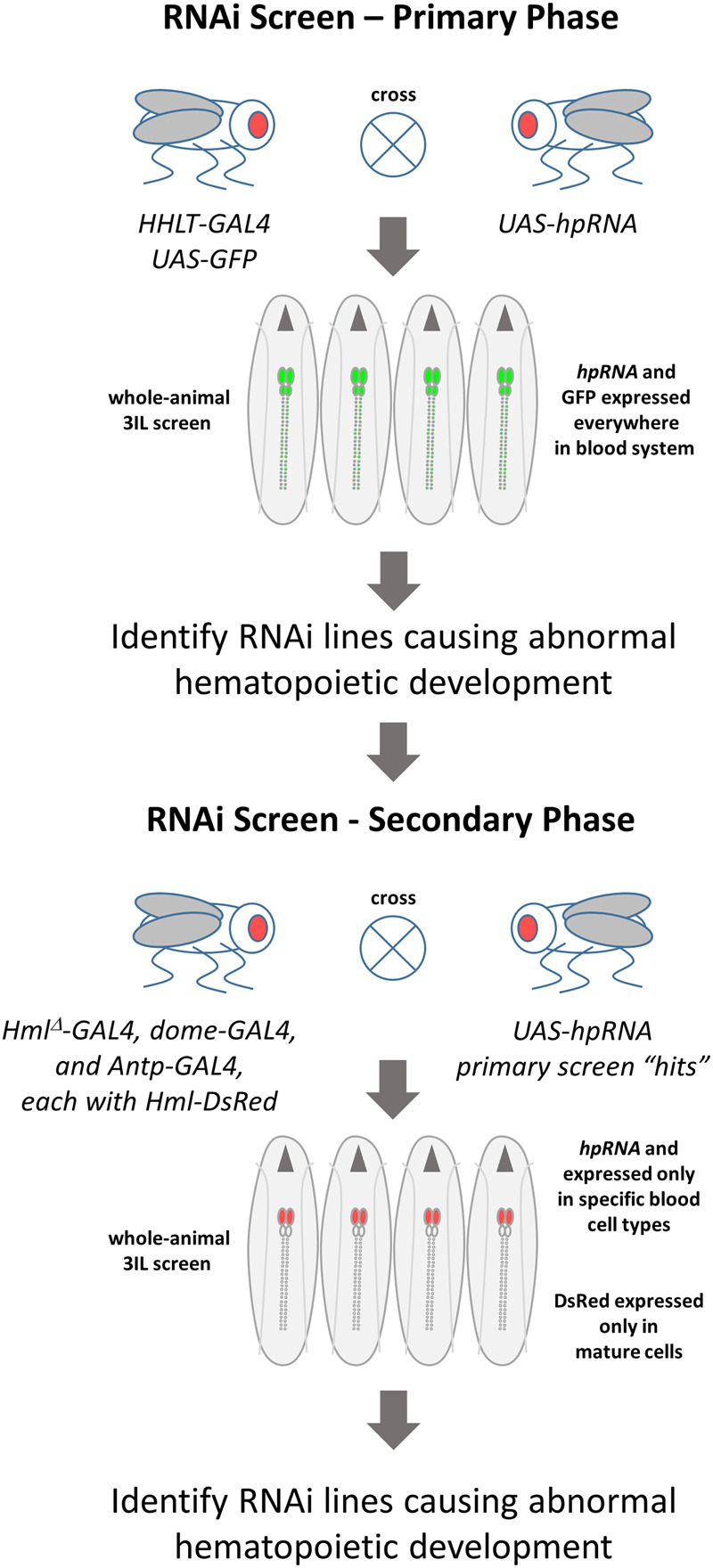
A functional genomics screen for new hematopoietic genes in *Drosophila*. In the primary screen, RNAi occurred throughout the larval hematopoietic system, which specifically expressed GFP. Briefly, *HHLT-GAL4 UAS-GFP* flies (Mondal *et al.* 2014) were crossed to flies carrying different *UAS-hairpin RNA* (*hpRNA*) transgenes targeting a unique gene. Progeny third-instar larvae expressed both hpRNAs (eliciting an RNAi response) and GFP throughout the blood system. Expression of GFP was monitored by fluorescence microscopy in whole larvae, four at a time. RNAi lines causing a discernable increase or decrease in GFP fluorescence, relative to control larvae lacking RNAi, were selected for use in the secondary screen. In the secondary screen, RNAi line “hits” from the primary screen were crossed to population-specific *GAL4* driver lines (*Hml^Δ^-GAL4* for maturing cells, *dome-GAL4* for progenitor cells, and *Antp-GAL4* for Posterior Signaling Center cells). These *GAL4* driver lines also carried *Hml^Δ^-DsRed* as a reporter of blood cell maturation. Expression of DsRed was monitored by fluorescence microscopy in whole larvae, four at a time.

**Figure 2:**
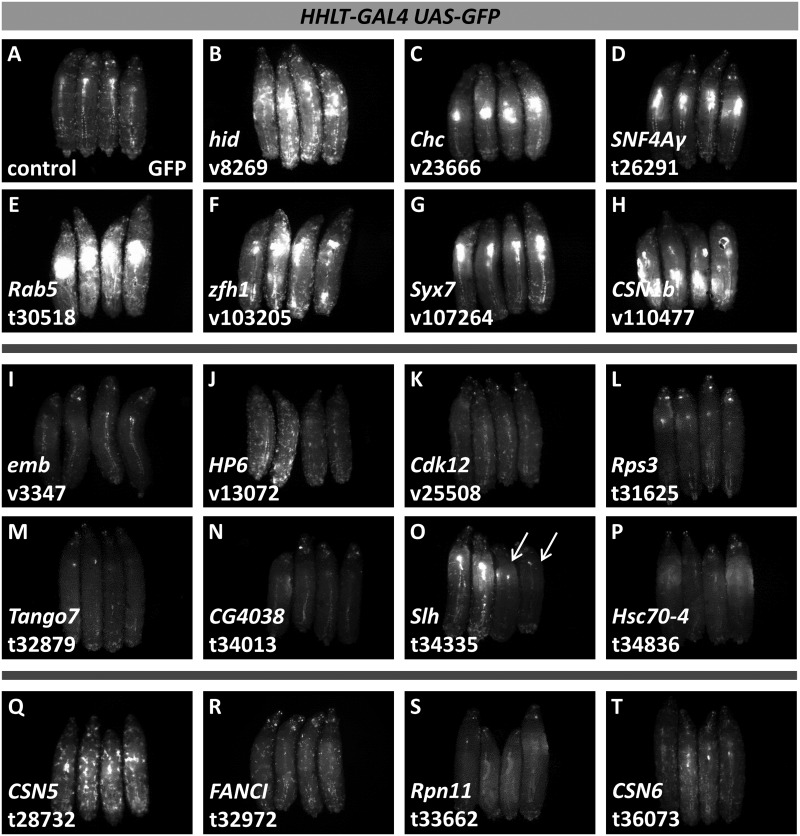
Select examples of candidate hematopoietic genes identified in the primary screen by *HHLT-GAL4 UAS-GFP* expression. For each image, four GFP-expressing, third-instar larvae are shown with anterior at the top and dorsal facing up. (A) control larvae lacking RNAi. (B)–(H) candidate genes that cause increased GFP fluorescence with RNAi. (I)–(P) candidate genes that cause decreased GFP fluorescence with RNAi. Note that in panel O, arrows point to larvae affected by RNAi; the unaffected sibling larvae arise because of heterozygosity of the *UAS-hpRNA* transgene in the parental line. (Q)–(T) candidate genes altering GFP expression in the circulating cell population. Target genes and specific RNAi lines are indicated in the lower left of the panel. Black spots observable in some larvae (e.g., panel H) are melanotic pseudotumors. RNAi line designations: v, VDRC; t, TRiP.

To identify hematopoietic genes, 339 URCFG students used RNA interference (RNAi) to disrupt the function of approximately 3500 genes within the developing blood system. In our experimental approach, pseudo-double-stranded hairpin RNAs (hpRNAs) are produced within cells from a transgene containing an inverted-repeat DNA sequence corresponding to a specific target gene (Ni *et al.* 2008). Subsequently, these hpRNAs are recognized and processed into an active RNA-induced silencing complex (RISC), initiating the RNAi response and the eventual degradation of target gene mRNAs (Mohr *et al.* 2014). Restriction of hpRNA production to blood cells was achieved by using the *GAL4*/*UAS* gene expression system derived from yeast (Elliott and Brand 2008). Students crossed *GAL4*-expressing lines with RNAi lines in which target-gene inverted-repeat sequences are under the control of the GAL4-responsive *UAS* enhancer. The primary RNAi screen made use of the *HHLT-GAL4* line (Mondal *et al.* 2014), in which GAL4 is expressed throughout the blood system. The *HHLT-GAL4* line also contains a *UAS-GFP* transgene, allowing for direct observation of the hematopoietic tissues (the lymph gland and circulating cells) in whole animals using fluorescence microscopy. An overview of the experimental design is shown in Figure 1. Using this line for screening over the course of several years, URCFG students ultimately identified 137 candidate genes (148 RNAi lines) involved in hematopoiesis (Table 1; see Figure 2 for examples).

**Table 1:** Identified genes causing a hematopoietic change when functionally disrupted (*HHLT-GAL4*)

#	RNAi line numeric ID	Library	Annotation symbol	Gene symbol	Gene name	GFP fluorescence— lymph gland	GFP fluorescence— circulation	GFP fluorescence —heart tube				
1	968	GD	CG1804	*kek6*	*kekkon 6*	Increased	Decreased	Missing				
2	3065	GD	CG10446	*Sidpn*	*similar to Deadpan*	Increasd +^b^	wt	wt				
3	6227	NIG	CG6227	*CG6227*	—	Decreased	wt	wt				
4	6819	NIG	CG6819	*mbo*	*members only*	Abnormal	wt	Missing				
5	7185	NIG	CG7185	*Cpsf6*	*Cleavage and polyadenylation specific factor 6*	Decreased	wt	wt				
6	7794	NIG	CG7794	*CG7794*	—	Decreased	Increased	Abnormal				
7	7819	GD	**CG15792**	***zip*** ^a^	***zipper***	Decreased	Decreased	wt				
8	8269	GD	CG5123	*hid*	*head involution defective*	Increased ++ /missing	Increased ++	wt				
9	9264	GD	CG8604	*Amph*	*Amphiphysin*	Decreased	Decreased	wt				
10	11152	GD	CG5505	*scny*	*scrawny*	Abnormal	wt	wt				
11	13072	GD	CG15636	*HP6*	*Heterochromatin protein 6*	Increased + / missing	Increased +	wt				
12	15565	GD	**CG4260**	***AP-2alpha***	***Adaptor protein complex 2, alpha subunit***	wt	wt	Abnormal				
13	17517	GD	CG14395	*CG14395*	—	Increased + / missing	wt	Decreased / missing				
14	23666	GD	**CG9012**	***Chc***	***Clathrin heavy chain***	Increased ++	wt	Decreased				
15	23772	GD	CG8730	*drosha*	*drosha*	Decreased	wt	Decreased				
16	24354	GD	CG2331	*TER94*	—	Missing	Decreased	Missing				
17	25427	GD	CG2677	*eIF2Bbeta*	*eukaryotic translation initiation factor 2B subunit beta*	Decreased/missing	Decreased	Decreased / missing				
18	25508	GD	CG7597	*Cdk12*	*Cyclin-dependent kinase 12*	Decreased/missing	Decreased/tumors	Decreased				
19	25811	VALIUM10	CG31136	*Syx1A*	*Syntaxin 1 A*	Decreased	wt	Missing				
20	25950	VALIUM10	CG11556	*Rph*	*Rabphilin*	Abnormal	Increased	wt				
21	25988	VALIUM10	CG2848	*Tnpo-SR*	*Transportin-Serine/Arginine rich*	Decreased	wt	wt				
22	26291	VALIUM10	CG17299	*SNF4Agamma*	*SNF4/AMP-activated protein kinase gamma subunit*	Increased	wt	wt				
23	26307	VALIUM10	CG3937	*cher*	*cheerio*	Missing^b^	Decreased	Missing				
24	26721	VALIUM10	CG13626	*Syx18*	*Syntaxin 18*	Decreased	Wt	Decreased / missing				
25	27299	GD	CG10663	*CG10663*	—	Decreased	Decreased	Missing				
26	27322	VALIUM10	CG6056	*AP-2sigma*	*Adaptor Protein complex 2, sigma subunit*	Increased	wt	wt				
27	27330	GD	CG10889	*CG10889*	—	Increased ++	Decreased	Missing				
28	27526	VALIUM10	CG8843	*Sec5*	*Secretory 5*	Increased +	wt	wt				
29	27530	VALIUM10	**CG9012**	***Chc***	***Clathrin heavy chain***	Increased ++ /missing	Increased ++/tumors	wt				
30	27553	VALIUM10	CG10174	*Ntf-2r*	*Nuclear transport factor-2-related*	Increased +	Increased ++	Abnormal				
31	27685	VALIUM10	CG33101	*Nsf2*	*N-ethylmaleimide-sensitive factor 2*	Decreased	Decreased	Missing				
32	28040	VALIUM10	CG7057	*AP-2mu*	*Adaptor Protein complex 2, mu subunit*	Increased ++ /missing	Increased ++/tumors	wt				
33	28047	VALIUM10	**CG8432**	***Rep***	***Rab escort protein***	Abnormal	Increased	Missing				
34	28329	VALIUM10	CG5686	*chico*	*chico*	Increased ++ / missing	Increased ++/tumors	Decreased / missing				
35	28342	VALIUM10	CG9575	*Rab35*	*Rab35*	Decreased	Decreased	wt				
36	28343	VALIUM10	CG8114	*pbl*	*pebble*	Missing^c^	Missing	Missing				
37	28513	VALIUM10	CG18102	*shi*	*shibire*	Increased +	Increased +	wt				
38	28621	VALIUM10	CG32547	*CG32547*	—	Decreased	wt	Missing				
39	28684	VALIUM10	CG43395	*Cngl*	*Cyclic nucleotide-gated ion channel-like*	Increased +	Decreased	Missing				
40	28712	VALIUM10	CG6095	*Exo84*	*Exocyst 84*	Decreased	Decreased	Decreased / missing				
41	28732	VALIUM10	CG14884	*CSN5*	*COP9 signalosome subunit 5*	Increased ++ / missing	Increased ++/tumors	wt				
42	28866	GD	**CG8432**	***Rep***	***Rab escort protein***	wt	wt	Decreased / missing				
43	28929	VALIUM10	CG15811	*Rop*	*Ras opposite*	Abnormal	Decreased	Missing				
44	29072	GD	CG9198	*shtd*	*shattered*	Decreased / missing	Decreased	wt				
45	29316	VALIUM10	CG8053	*eIF1A*	*eukaryotic translation initiation factor 1 A*	Missing	Decreased	Missing				
46	29385	VALIUM10	CG10149	*Rpn6*	*Regulatory particle non-ATPase 6*	Decreased / missing	Decreased	Missing				
47	29520	VALIUM10	CG1877	*Cul1*	*Cullin 1*	Abnormal	wt	Decreased / missing				
48	29535	VALIUM10	CG3193	*crn*	*crooked neck*	Decreased / missing	Decreased	Decreased / missing				
49	29575	GD	CG1957	*Cpsf100*	*Cleavage and polyadenylation specificity factor 100*	Decreased	Decreased/tumors	wt				
50	29587	VALIUM10	**CG6625**	***alphaSnap***	***alpha Soluble NSF attachment protein***	wt	Decreased	Missing				
51	29741	GD	CG33507	*dpr2*	*defective proboscis extension response 2*	Decreased	wt	Decreased / missing				
52	30515	VALIUM10	CG4654	*Dp*	*DP transcription factor*	Decreased	wt	wt				
53	30518	VALIUM10	CG3664	*Rab5*	*Rab5*	Increased ++	Increased ++	wt				
54	31090	VALIUM1	CG8954	*Smg5*	*Smg5*	Increased +	Increased +	wt				
55	31196	VALIUM1	**CG11092**	***Nup93-1***	***Nucleoporin 93kD-1***	Decreased	Decreased	Missing				
56	31765	VALIUM1	CG9652	*Dop1R1*	*Dopamine 1-like receptor 1*	Decreased	wt	wt				
57	31893	VALIUM10	CG7178	*wupA*	*wings up A*	Decreased/missing	Low, clump	Decreased / missing				
58	32365	VALIUM20	CG1250	*Sec23*	*Secretory 23*	Missing	Low	Missing				
59	32369	VALIUM20	CG10212	*SMC2*	*Structural maintenance of chromosomes 2*	Decreased/missing	wt	Decreased / missing				
60	32415	VALIUM20	CG9750	*rept*	*reptin*	Missing	Decreased	Missing				
61	32503	VALIUM20	**CG4303**	***Bap60***	***Brahma associated protein 60kD***	Decreased	Decreased	wt				
62	32510	VALIUM20	CG7420	*CG7420*	—	Missing	Decreased	Missing				
63	32854	VALIUM20	CG5374	*CCT1*	*Chaperonin containing TCP1 subunit 1*	Decreased/missing	Increased +	Decreased / missing				
64	32865	VALIUM20	CG5519	*Prp19*	*Pre-RNA processing factor 19*	Decreased/missing	Decreased	wt				
65	32866	VALIUM20	**CG4260**	***AP-2alpha***	***Adaptor Protein complex 2, alpha subunit***	Increased +	Increased ++	Decreased / missing				
66	32877	VALIUM20	CG12264	*Nfs1*	*Nfs1 cysteine desulfurase*	Decreased/missing	Decreased	Abnormal				
67	32879	VALIUM20	**CG8309**	***eIF3m***	***eukaryotic translation initiation factor 3 subunit m***	Decreased	Decreased	Abnormal				
68	32972	VALIUM20	CG13745	*FANCI*	*Fanconi anemia complementation group I*	Decreased	Decreased	Decreased / missing				
69	32989	VALIUM20	CG7581	*Bub3*	*Bub3*	Decreased/missing	wt	wt				
70	33003	VALIUM20	CG11856	*Nup358*	*Nucleoporin 358kD*	Missing	Decreased	Missing				
71	33043	VALIUM20	CG9193	*PCNA*	*Proliferating cell nuclear antigen*	Decreased	Decreased	Decreased / missing				
72	33615	VALIUM20	CG4006	*Akt1*	*Akt1*	Missing	Decreased	Missing				
73	33655	VALIUM20	CG9745	*D1*	*D1 chromosomal protein*	Abnormal	Increased +	Decreased / missing				
74	33660	VALIUM20	CG1519	*Prosalpha7*	*Proteasome alpha7 subunit*	Missing	Decreased	Missing				
75	33662	VALIUM20	CG18174	*Rpn11*	*Regulatory particle non-ATPase 11*	Missing	Decreased	Missing				
76	33725	VALIUM20	CG7471	*HDAC1*	*Histone deacetylase 1*	Decreased	wt	wt				
77	33727	VALIUM20	CG6671	*AGO1*	*Argonaute-1*	Missing	Decreased	wt				
78	33897	VALIUM20	CG3820	*Nup214*	*Nucleoporin 214kD*	Missing	Decreased	Missing				
79	33908	VALIUM20	**CG11092**	***Nup93-1***	***Nucleoporin 93kD-1***	Increased +	wt	wt				
80	33954	VALIUM20	**CG4303**	***Bap60***	***Brahma associated protein 60kD***	Decreased/missing	Low	wt				
81	33986	VALIUM20	CG3412	*slmb*	*supernumerary limbs*	Decreased/missing	Decreased	Missing				
82	34013	VALIUM20	CG4038	*CG4038*	—	Missing	Decreased	Decreased / missing				
83	34074	VALIUM20	CG8728	*CG8728*	—	Decreased/missing	wt	Decreased / missing				
84	34090	VALIUM20	**CG11092**	***Nup93-1***	***Nucleoporin 93kD-1***	Missing	Decreased	Missing				
85	34335	VALIUM20	CG3539	*Slh*	*SLY-1 homologous*	Decreased	Decreased	Missing				
86	34339	VALIUM20	CG5660	*ValRS-m*	*Valyl-tRNA synthetase, mitochondrial*	Decreased	Decreased	wt				
87	34356	VALIUM20	CG5706	*beta-PheRS*	*Phenylalanyl-tRNA synthetase, beta-subunit*	Decreased/missing	wt	Decreased / missing				
88	34359	VALIUM20	CG6877	*Atg3*	*Autophagy-related 3*	Abnormal	Decreased	Missing				
89	34483	VALIUM20	CG33123	*LeuRS*	*Leucyl-tRNA synthetase*	Decreased / missing	Decreased	Decreased / missing				
90	34551	VALIUM20	CG10483	*CG10483*	—	Decreased	Decreased	wt				
91	34567	VALIUM20	CG7885	*RpII33*	*RNA polymerase II 33kD subunit*	Missing	Decreased	Missing				
92	34582	VALIUM20	CG3762	*Vha68-2*	*Vacuolar H[+] ATPase 68 kDa subunit 2*	Abnormal	wt	Decreased / missing				
93	34626	VALIUM20	CG1101	*Ref^1^*	*RNA and export factor binding protein 1*	Increased +	wt	wt				
94	34685	VALIUM20	CG6783	*fabp*	*fatty acid binding protein*	Abnormal	Clump	Abnormal				
95	34705	VALIUM20	CG4717	*kni*	*knirps*	Decreased/missing	Decreased	Missing				
96	34711	GD	CG3806	*eIF2Bepsilon*	*eukaryotic translation initiation factor 2B subunit epsilon*	Decreased/missing	wt	wt				
97	34727	GD	**CG3889**	***CSN1b***	***COP9 signalosome subunit 1 b***	Increased ++ / missing	Increased ++ / tumors	Decreased / missing				
98	34730	VALIUM20	CG2051	*Hat1*	*Histone acetyltransferase 1*	wt	Decreased	Missing				
99	34788	VALIUM20	CG11985	*Sf3b5*	*Splicing factor 3 b subunit 5*	Missing	Decreased	Missing				
100	34836	VALIUM20	CG4264	*Hsc70-4*	*Heat shock protein cognate 4*	Decreased/missing	Decreased	Decreased / missing				
101	34840	VALIUM20	CG16941	*Sf3a1*	*Splicing factor 3a subunit 1*	Missing	Decreased	Missing				
102	34857	VALIUM20	CG10333	*CG10333*	—	Decreased/missing	Decreased	wt				
103	34860	VALIUM20	CG11920	*CG11920*	—	Decreased	Decreased	Decreased / missing				
104	34876	VALIUM20	CG1430	*bys*	*by S6*	Missing	wt	Missing				
105	34969	VALIUM20	CG8977	*CCT3*	*Chaperonin containing TCP1 subunit 3*	Decreased/missing	wt	wt				
106	34982	VALIUM20	CG5179	*Cdk9*	*Cyclin-dependent kinase 9*	wt	wt	Decreased / missing				
107	35741	VALIUM20	CG5429	*Atg6*	*Autophagy-related 6*	Increased +	wt	wt				
108	35986	GD	CG8610	*Cdc27*	*Cell division cycle 27*	Abnormal	wt	Missing				
109	36073	VALIUM22	CG6932	*CSN6*	*COP9 signalosome subunit 6*	Increased ++/missing	Increased ++/tumors	wt				
110	36113	VALIUM20	CG6699	*beta'COP*	*Coat Protein (coatomer) beta'*	Missing	Decreased	Missing				
111	36727	VALIUM20	**CG15792**	***zip***	***zipper***	Decreased	wt	wt				
112	43116	GD	CG6998	*ctp*	*cut up*	Increased +	wt	wt				
113	44288	GD	CG9033	*Tsp47F*	*Tetraspanin 47 F*	Decreased	wt	wt				
114	45027	GD	CG5605	*eRF1*	*eukaryotic translation release factor 1*	Missing	Increased ++/tumors	Missing				
115	46554	GD	CG17369	*Vha55*	*Vacuolar H[+]-ATPase 55kD subunit*	Decreased	wt-high	Decreased / missing				
116	48044	GD	CG9556	*alien*	*alien*	Increased ++/missing	Increased ++	wt				
117	100545	KK	CG2788	*Ugt36A1*	*UDP-glycosyltransferase family 36 member A1*	Decreased	wt	wt				
118	100749	KK	CG8639	*Cirl*	*Calcium-independent receptor for alpha-latrotoxin*	Increased +	Increased +	wt				
119	101248	KK	CG7051	*Dic61B*	*Dynein intermediate chain at 61B*	wt	Increased +	wt				
120	101341	KK	**CG6625**	***alphaSnap***	***alpha Soluble NSF attachment protein***	Abnormal	wt	Dcreased / missing				
121	101404	KK	CG44436	*sno*	*strawberry notch*	Increased +	Increased ++	wt				
122	101513	KK	CG3186	*eEF5*	*eukaryotic translation elongation factor 5*	Decreased/missing	wt	Decreased / missing				
123	102406	KK	CG2216	*Fer1HCH*	*Ferritin 1 heavy chain homologue*	Increased ++/missing	Increased ++/tumors	missing				
124	103205	KK	CG1322	*zfh1*	*Zn finger homeodomain 1*	Increased ++/missing	Increased ++	wt				
125	103383	KK	**CG9012**	***Chc***	***Clathrin heavy chain***	Increased +	Decreased/tumors	Decreased / missing				
126	103557	KK	CG6177	*ldlCp*	*ldlCp-related protein*	wt	wt	Decreased / missing				
127	103661	KK	CG42611	*mgl*	*Megalin*	wt	Increased	wt				
128	103704	KK	CG1560	*mys*	*myospheroid*	Decreased	Decreased	missing				
129	103767	KK	CG13387	*emb*	*embargoed*	Increased ++/missing	Increased ++	Decreased / missing				
130	104210	KK	CG7000	*Snmp1*	*Sensory neuron membrane protein 1*	Increased ++/missing	Increased ++	Decreased / missing				
131	105325	KK	CG8636	*eIF3g1*	*eukaryotic translation initiation factor 3 subunit g1*	Decreased	Decreased	Decreased / missing				
132	105653	KK	CG2095	*Sec8*	*Secretory 8*	Increased +	wt	Decreased / missing				
133	105706	KK	CG18247	*Shark*	*SH2 ankyrin repeat kinase*	Abnormal	Decreased	Decreased / missing				
134	105763	KK	CG17737	*eIF1*	*eukaryotic translation initiation factor 1*	Increased ++/missing	Increased ++	Decreased / missing				
135	105836	KK	CG5341	*Sec6*	*Secretory 6*	Increased +	Increased +	Decreased / missing				
136	106144	KK	CG6094	*CG6094*	—	Increased ++/missing	Increased ++	Decreased / missing				
137	106240	KK	CG6382	*eRF3*	*eukaryotic translation release factor 3*	Increased ++/missing	Increased ++	Decreased / missing				
138	107264	KK	CG5081	*Syx7*	*Syntaxin 7*	Increased +	wt	wt				
139	107268	KK	CG2238	*eEF2*	*eukaryotic translation elongation factor 2*	Decreased/abnormal	wt	Missing				
140	107277	KK	CG5371	*RnrL*	*Ribonucleoside diphosphate reductase large subunit*	Increased ++/missing	Increased ++	Missing				
141	107622	KK	CG2637	*Fs(2)Ket*	*Female sterile (2) Ketel*	Missing	Increased ++	Missing				
142	108415	KK	CG7935	*msk*	*moleskin*	Abnormal	wt	Decreased / missing				
143	109280	KK	CG9191	*Klp61F*	*Kinesin-like protein at 61 F*	Abnormal	wt	wt				
144	109782	KK	CG10840	*eIF5B*	*eukaryotic translation initiation factor 5B*	Missing	Decreased/tumors	Missing				
145	110355	KK	CG7831	*ncd*	*non-claret disjunctional*	Abnormal	Increased ++	wt				
146	110359	KK	**CG8309**	***eIF3m***	***eukaryotic translation initiation factor 3 subunit m***	Decreased	Decreased	Missing				
147	110477	KK	**CG3889**	***CSN1b***	***COP9 signalosome subunit 1 b***	Increased ++/missing	Increased ++/tumors	wt				
148	110774	KK	CG15218	*CycK*	*Cyclin K*	Increased +	Increased +	wt				

a Genes in bold font were identified more than once.

b Increased +: strongly increased/enlarged; increased ++: very strongly increased/enlarged; increased/missing: extreme increase/enlargement with disintegration.

c May carry a balancer chromosome.

### Cell-type specific RNAi and the effect on blood cell maturation

The primary RNAi screen with *HHLT-GAL4 UAS-2XEGFP* was useful in identifying candidate hematopoietic genes due to the relative ease of discerning gross defects in the lymph gland and the circulating blood cells through changes in GFP fluorescence. However, this screen could neither indicate a cell-type-specific function for the identified gene (as *HHLT-GAL4* is expressed in mature, progenitor, and signaling cells) nor what the specific impact was on blood lineage development. To address these limitations and further delineate the functions of the identified candidate genes, the secondary screen was conducted in which RNAi was directed to either differentiating cells using the *Hemolectin^Δ^-GAL4* (*Hml^Δ^-GAL4*; Sinenko and Mathey-Prevot 2004; Jung *et al.* 2005), or progenitor cells using *domeless-GAL4* (*dome^PG14^-GAL4* or the derivative *dome^MESO^-GAL4*; Jung *et al.* 2005; Yoon *et al.* 2017), or PSC cells using *Antennapedia-GAL4* (*Antp-GAL4*; Mandal *et al.* 2007). Each of these secondary-screen *GAL4* driver lines also carried *Hml^Δ^-DsRed* (Makhijani *et al.* 2011) as a marker of hematopoietic maturation and to facilitate screening in whole animals. In this way, candidate genes with developmental roles in specific blood cell populations could be identified.

We compiled a collection of 202 RNAi lines comprised of the 148 lines identified in the primary screen, as well as 54 lines (Supplementary Table S1) that target either the primary screen candidate genes redundantly (20 genes) or genes predicted to function in related processes or pathways. Over the course of five academic quarters (Winter 2015–Spring 2016), students crossed RNAi lines from the 202-line collection with the three *GAL4* drivers described above and analyzed DsRed fluorescence (*Hml^Δ^-DsRed* expression) in whole, wandering third-instar larvae. Each RNAi line was assigned to two or more students, with the goal of collecting at least two complete data sets for each *GAL4* driver/RNAi line cross combination. The collection of imaging data for 12 progeny larvae from a given cross was considered a complete data set, and individual RNAi lines remained within the assignment pool until two complete image data sets were obtained. The duplicate completion rate for the entire RNAi line collection was 41% (83 lines) for all three GAL4 drivers, 78% (158 lines) for at least two of three GAL4 drivers, and 95% (191 lines (95%) for at least one GAL4 driver. If single-complete data sets are included, the completion rate increases to 75% (151 lines) across all three drivers, to 99% (199 lines) for at least two of three GAL4 drivers, and to 100% (202 lines) for at least one GAL4 driver (Supplementary Table S2). With respect to RNAi in the PSC (*Hml^Δ^-DsRed*; *Antp-GAL4* with or without *elav-GAL80*; Rideout *et al.* 2010), 188 of the 202 RNAi lines were analyzed (93%), eight of which were found to be lethal (presumably due to GAL4 activity outside of the lymph gland that is not suppressed by *elav-GAL80*). Of the 180 viable lines, 160 (79%) were completed in duplicate. For RNAi screening in progenitor cells (*Hml^Δ^-DsRed*; *dome-GAL4* or *elav-GAL80*; *Hml^Δ^-DsRed*; *dome^MESO^-GAL4*), students successfully screened 186 RNAi lines (of 202; 92%), of which 137 (68%) were completed in duplicate. Similar screening in the maturing blood cell population (*Hml^Δ^-DsRed Hml^Δ^-GAL4*) was successful for 182 RNAi lines (of 202; 90%), 135 (67%) of which were completed in duplicate (Supplementary Table S2).

As described previously, phenotypic analysis in the secondary screen involved discerning the variance of *Hml^Δ^-DsRed* reporter expression between RNAi and control (non-RNAi) backgrounds as viewed in whole, third instar larvae. While this is a highly specific and, therefore, powerful molecular genetic tool, the usefulness of *Hml^Δ^-DsRed* in this RNAi screen is offset by variability in lymph gland phenotypes, possibly due to incomplete phenotypic penetrance and/or expressivity, within the 12 RNAi-larvae sample group. Additionally, the relative inexperience of the undergraduate researchers, with *Drosophila* in general and the hematopoietic system in particular, sometimes made their identification of subtle phenotypic changes difficult. Therefore, to increase the likelihood that RNAi lines (candidate genes) are identified correctly, the developmental phenotype caused by each RNAi line was independently scored by two or more students. Scoring consisted of first determining whether a phenotype was present and, if so, then describing and categorizing the nature of the phenotype. RNAi lines identified more than once and causing similar hematopoietic phenotypes (the majority of lines identified) were subdivided into those causing an increase in lymph gland *Hml^Δ^-DsRed* expression and those causing a decrease in lymph gland *Hml^Δ^-DsRed* expression. Though not our focus, changes in *Hml^Δ^-DsRed* expression among circulating cells were also noted (the vast majority of which also had a lymph gland phenotype; Supplementary Table S3).

**Figure 3:**
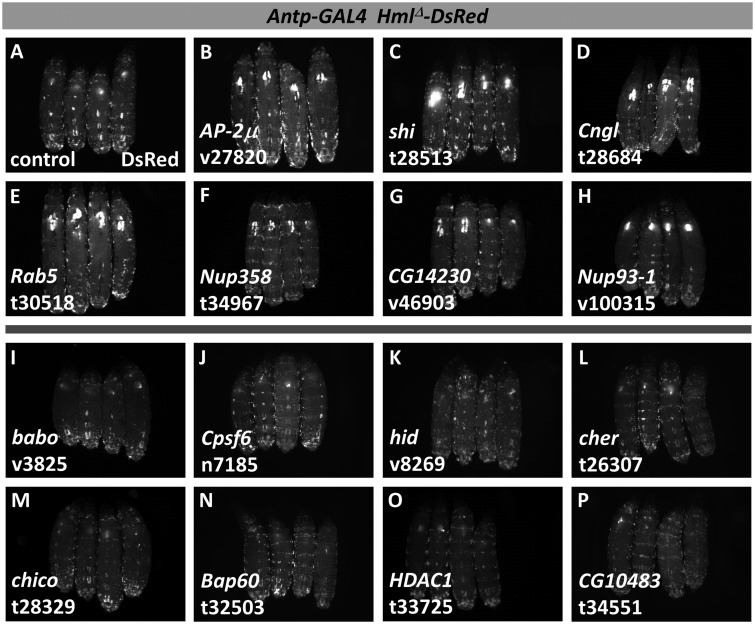
Select examples of candidate hematopoietic genes identified in the secondary screen by *Antp-GAL4 Hml^Δ^-DsRed* expression. For each image, four DsRed-expressing, third-instar larvae are shown with anterior at the top and dorsal facing up. (A) control larvae lacking RNAi. (B–H) candidate genes that cause increased DsRed fluorescence with RNAi. (I–P) candidate genes that cause decreased DsRed fluorescence with RNAi. Target genes and specific RNAi lines are indicated in the lower left of the panel. RNAi line designations: v, VDRC; t, TRiP; n, NIG.

**Figure 4:**
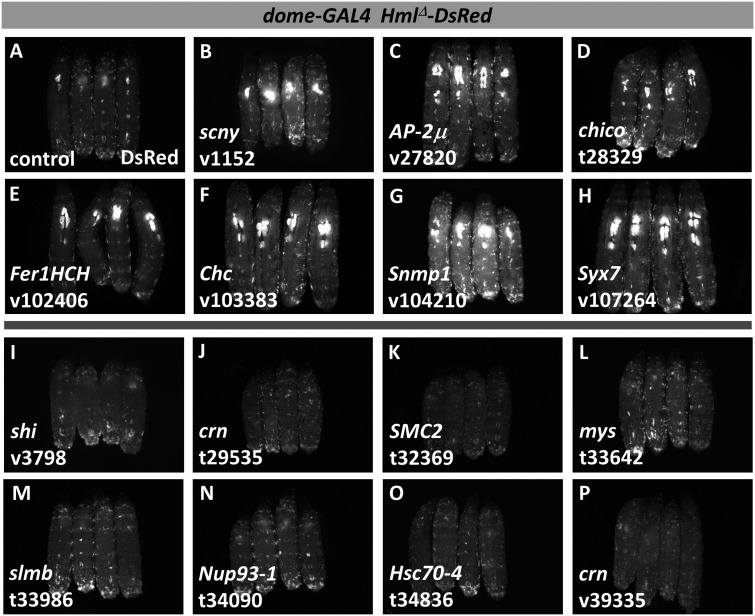
Select examples of candidate hematopoietic genes identified in the secondary screen by *dome-GAL4 Hml^Δ^-DsRed* expression. For each image, four DsRed-expressing, third-instar larvae are shown with anterior at the top and dorsal facing up. (A) control larvae lacking RNAi. (B–H) candidate genes that cause increased DsRed fluorescence with RNAi. (I–P) candidate genes that cause decreased DsRed fluorescence with RNAi. Target genes and specific RNAi lines are indicated in the lower left of the panel. RNAi line designations: v, VDRC; t, TRiP.

**Figure 5:**
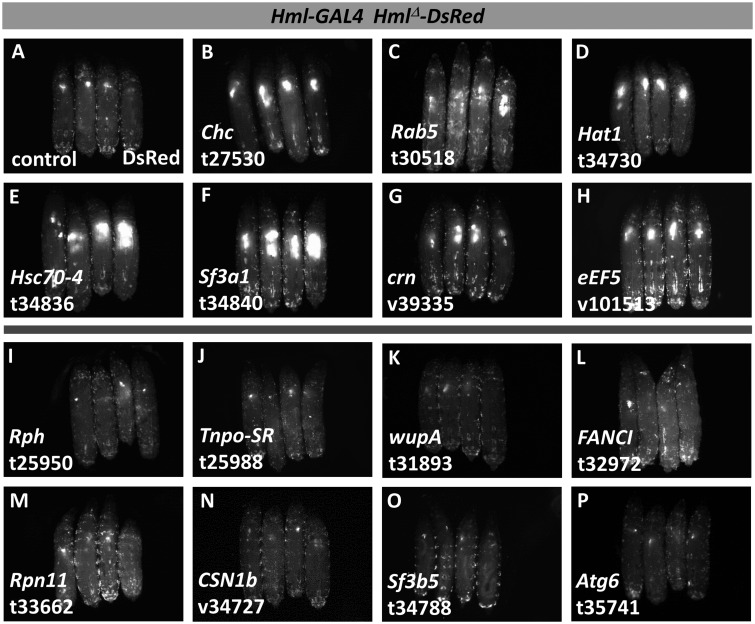
Select examples of candidate hematopoietic genes identified in the secondary screen by *Hml^Δ^-GAL4 Hml^Δ^-DsRed* expression. For each image, four DsRed-expressing, third-instar larvae are shown with anterior at the top and dorsal facing up. (A) control larvae lacking RNAi. (B–H) candidate genes that cause increased DsRed fluorescence with RNAi. (I–P) candidate genes that cause decreased DsRed fluorescence with RNAi. Target genes and specific RNAi lines are indicated in the lower left of the panel. RNAi line designations: v, VDRC; t, TRiP.

Directing RNAi to the PSC using *Antp-GAL4* identified 20 RNAi lines (representing 19 genes) that cause an increase in *Hml^Δ^-DsRed* lymph gland fluorescence and 15 RNAi lines (representing 15 genes) that cause a decrease in *Hml^Δ^-DsRed* lymph gland fluorescence (see Figure 3 for examples; Table 2). This analysis also identified 13 RNAi lines (representing 13 genes) associated with a change in the circulating cell population (Supplementary Table S3). Of these 13 RNAi lines, three overlap with RNAi lines increasing lymph gland DsRed fluorescence and four overlap with RNAi lines decreasing lymph gland DsRed fluorescence. The use of *dome-GAL4* to disrupt gene function in lymph gland progenitor cells identified 34 RNAi lines (representing 33 genes) increasing lymph gland *Hml^Δ^-DsRed* expression and 18 RNAi lines (representing 17 genes) decreasing lymph gland *Hml^Δ^-DsRed* expression (see Figure 4 for examples; Table 3). Another 17 RNAi lines (representing 16 genes) were identified that cause a change in the circulating blood cell population (Supplementary Table S3), with six lines in common with those increasing lymph gland DsRed fluorescence and six lines in common with those decreasing lymph gland DsRed fluorescence. Lastly, analysis of RNAi lines in maturing/mature cells using *Hml^Δ^-GAL4* identified 50 RNAi lines (representing 48 genes) causing an increase in lymph gland *Hml^Δ^-DsRed* expression and 8 RNAi lines (representing 8 genes) causing a decrease in lymph gland *Hml^Δ^-DsRed* expression (see Figure 5 for examples; Table 4). A total of 38 RNAi lines (representing 36 genes) caused a change in the circulating cell population (Supplementary Table S3), with 27 lines overlapping with those causing an increase in lymph gland *Hml^Δ^-DsRed* fluorescence and four lines overlapping with those causing a decrease in lymph gland *Hml^Δ^-DsRed* fluorescence.

**Table 2:** Genes causing a change in lymph gland *Hml*^*Δ*^*-DsRed* expression when disrupted in PSC cells (*Antp-GAL4*)

#	Numeric ID	Library	Annotation symbol	Gene symbol	Gene name	**Change in *Hml*** ^*Δ*^ ***-DsRed***
1	27820	GD	CG7057	*AP-2mu*	*Adaptor Protein complex 2, mu subunit*	Increase
2	28513	VALIUM10	CG18102	*shi*	*Shibire*	Increase
3	28684	VALIUM10	CG43395	*Cngl*	*Cyclic nucleotide-gated ion channel-like*	Increase
4	29072	GD	CG9198	*shtd*	*Shattered*	Increase
5	30518	VALIUM10	CG3664	*Rab5*	*Rab5*	Increase
6	32415	VALIUM20	CG9750	*rept*	*Reptin*	Increase
7	33655	VALIUM20	CG9745	*D1*	*D1 chromosomal protein*	Increase
8	34840	VALIUM20	CG16941	*Sf3a1*	*Splicing factor 3a subunit 1*	Increase
9	34967	VALIUM20	CG11856	*Nup358*	*Nucleoporin 358kD*	Increase
10	36583	VALIUM22	CG7507	*Dhc64C*	*Dynein heavy chain 64 C*	Increase
11	46903	GD	CG14230	***CG14230*** ^a^	—	Increase
12	100315	KK	CG11092	*Nup93-1*	*Nucleoporin 93kD-1*	Increase
13	101531	KK	CG1133	*opa*	*odd paired*	Increase
14	102406	KK	CG2216	*Fer1HCH*	*Ferritin 1 heavy chain homologue*	Increase
15	103383	KK	CG9012	*Chc*	*Clathrin heavy chain*	Increase
16	103557	KK	CG6177	*ldlCp*	*ldlCp-related protein*	Increase
17	104096	KK	CG14230	***CG14230***	—	Increase
18	104210	KK	CG7000	*Snmp1*	*Sensory neuron membrane protein 1*	Increase
19	105653	KK	CG2095	*Sec8*	*Secretory 8*	Increase
20	108947	KK	CG17492	*mib2*	*mind bomb 2*	Increase
1	3825	GD	CG8224	*babo*	*baboon*	Decrease
2	7185	NIG	CG7185	*Cpsf6*	*Cleavage and polyadenylation specific factor 6*	Decrease
3	8269	GD	CG5123	*hid*	*head involution defective*	Decrease
4	25427	GD	CG2677	*eIF2Bbeta*	*eukaryotic translation initiation factor 2B subunit beta*	Decrease
5	26307	VALIUM10	CG3937	*cher*	*cheerio*	Decrease
6	28329	VALIUM10	CG5686	*chico*	*chico*	Decrease
7	28712	VALIUM10	CG6095	*Exo84*	*Exocyst 84*	Decrease
8	29741	GD	CG33507	*dpr2*	*defective proboscis extension response 2*	Decrease
9	32503	VALIUM20	CG4303	*Bap60*	*Brahma associated protein 60kD*	Decrease
10	33725	VALIUM20	CG7471	*HDAC1*	*Histone deacetylase 1*	Decrease
11	34551	VALIUM20	CG10483	*CG10483*	—	Decrease
12	34865	VALIUM20	CG7008	*Tudor-SN*	*Tudor Staphylococcal nuclease*	Decrease
13	37609	GD	CG7583	*CtBP*	*C-terminal binding protein*	Decrease
14	39335	GD	CG3193	*crn*	*crooked neck*	Decrease
15	45027	GD	CG5605	*eRF1*	*eukaryotic translation release factor 1*	Decrease

a Genes in bold font were identified more than once.

**Table 3:** Genes causing a change in lymph gland *Hml*^*Δ*^-*DsRed* expression when disrupted in immature blood cells (*dome*-*GAL4*)

#	Numeric ID	Library	Annotation symbol	Gene symbol	Gene name	**Change in *Hml*** ^*Δ*^ ***-DsRed***	
1	3065	GD	CG10446	*Sidpn*	*similar to Deadpan*	Increase	
2	9489	GD	CG2788	*Ugt36A1*	*UDP-glycosyltransferase family 36 member A1*	Increase	
3	11152	GD	CG5505	*scny*	*scrawny*	Increase	
4	22308	GD	CG6932	*CSN6*	*COP9 signalosome subunit 6*	Increase	
5	27553	VALIUM10	CG10174	*Ntf-2r*	*Nuclear transport factor-2-related*	Increase	
6	27820	GD	CG7057	*AP-2mu*	*Adaptor Protein complex 2, mu subunit*	Increase	
7	28329	VALIUM10	CG5686	*chico*	*chico*	Increase	
8	28732	VALIUM10	CG14884	*CSN5*	*COP9 signalosome subunit 5*	Increase	
9	29072	GD	CG9198	*shtd*	*shattered*	Increase	
10	30518	VALIUM10	CG3664	*Rab5*	*Rab5*	Increase	
11	32972	VALIUM20	CG13745	*FANCI*	*Fanconi anemia complementation group I*	Increase	
12	33043	VALIUM20	CG9193	*PCNA*	*Proliferating cell nuclear antigen*	Increase	
13	33655	VALIUM20	CG9745	*D1*	*D1 chromosomal protein*	Increase	
14	33660	VALIUM20	CG1519	*Prosalpha7*	*Proteasome alpha7 subunit*	Increase	
15	34582	VALIUM20	CG3762	*Vha68-2*	*Vacuolar* *H[+]* *ATPase 68 kDa subunit 2*	Increase	
16	34840	VALIUM20	CG16941	*Sf3a1*	*Splicing factor 3a subunit 1*	Increase	
17	34982	VALIUM20	CG5179	*Cdk9*	*Cyclin-dependent kinase 9*	Increase	
18	37609	GD	CG7583	*CtBP*	*C-terminal binding protein*	Increase	
19	46903	GD	CG14230	***CG14230*** ^a^	—	Increase	
20	48044	GD	CG9556	*alien*	*alien*	Increase	
21	100693	KK	CG11901	*eEF1gamma*	*eukaryotic translation elongation factor 1 gamma*	Increase	
22	101404	KK	CG44436	*sno*	*strawberry notch*	Increase	
23	101513	KK	CG3186	*eEF5*	*eukaryotic translation elongation factor 5*	Increase	
24	102406	KK	CG2216	*Fer1HCH*	*Ferritin 1 heavy chain homologue*	Increase	
25	103205	KK	CG1322	*zfh1*	*Zn finger homeodomain 1*	Increase	
26	103383	KK	CG9012	*Chc*	*Clathrin heavy chain*	Increase	
27	103557	KK	CG6177	*ldlCp*	*ldlCp-related protein*	Increase	
28	104096	KK	CG14230	***CG14230***	—	Increase	
29	104210	KK	CG7000	*Snmp1*	*Sensory neuron membrane protein 1*	Increase	
30	106240	KK	CG6382	*eRF3*	*eukaryotic translation release factor 3*	Increase	
31	107264	KK	CG5081	*Syx7*	*Syntaxin 7*	Increase	
32	107268	KK	CG2238	*eEF2*	*eukaryotic translation elongation factor 2*	Increase	
33	109280	KK	CG9191	*Klp61F*	*Kinesin-like protein at 61 F*	Increase	
34	110359	KK	CG8309	*eIF3m*	*eukaryotic translation initiation factor 3 subunit m*	Increase	
1	3798	GD	CG18102	*shi*	*shibire*	Decrease	
2	6227	NIG	CG6227	*CG6227*	—	Decrease	
3	7185	NIG	CG7185	*Cpsf6*	*Cleavage and polyadenylation specific factor 6*	Decrease	
4	7819	GD	CG15792	*zip*	*zipper*	Decrease	
5	29535	VALIUM10	CG3193	***crn***	***crooked neck***	Decrease	
6	31625	VALIUM1	CG6779	*RpS3*	*Ribosomal protein S3*	Decrease	
7	32365	VALIUM20	CG1250	*Sec23*	*Secretory 23*	Decrease	
8	32369	VALIUM20	CG10212	*SMC2*	*Structural maintenance of chromosomes 2*	Decrease	
9	32865	VALIUM20	CG5519	*Prp19*	*Pre-RNA processing factor 19*	Decrease	
10	33642	VALIUM20	CG1560	*mys*	*myospheroid*	Decrease	
11	33986	VALIUM20	CG3412	*slmb*	*supernumerary limbs*	Decrease	
12	34074	VALIUM20	CG8728	*CG8728*	—	Decrease	
13	34090	VALIUM20	CG11092	*Nup93-1*	*Nucleoporin 93kD-1*	Decrease	
14	34356	VALIUM20	CG5706	*beta-PheRS*	*Phenylalanyl-tRNA synthetase, beta-subunit*	Decrease	
15	34836	VALIUM20	CG4264	*Hsc70-4*	*Heat shock protein cognate 4*	Decrease	
16	34860	VALIUM20	CG11920	*CG11920*	—	Decrease	
17	39335	GD	CG3193	***crn***	***crooked neck***	Decrease	
18	40865	VALIUM20	CG5505	*scny*	*scrawny*	Decrease	

a Genes in bold font were identified more than once.

**Table 4:** Genes causing a change in lymph gland *Hml*^*Δ*^*-DsRed* expression when disrupted in mature blood cells (*Hml*^*Δ*^*-GAL4*)

#	Numeric ID	Library	Annotation symbol	Gene symbol	Gene name	**Change in *Hml*** ^*Δ*^ ***-DsRed***
1	3798	GD	CG18102	*shi*	*shibire*	Increase
2	7185	NIG	CG7185	*Cpsf6*	*Cleavage and polyadenylation specific factor 6*	Increase
3	11152	GD	CG5505	*scny*	*scrawny*	Increase
4	13072	GD	CG15636	*HP6*	*Heterochromatin protein 6*	Increase
5	15565	GD	CG4260	*AP-2alpha*	*Adaptor protein complex 2, alpha subunit*	Increase
6	17517	GD	CG14395	*CG14395*	—	Increase
7	23666	GD	CG9012	***Chc***	***Clathrin heavy chain*** ^a^	Increase
8	25811	VALIUM10	CG31136	*Syx1A*	*Syntaxin 1 A*	Increase
9	27322	VALIUM10	CG6056	*AP-2sigma*	*Adaptor Protein complex 2, sigma subunit*	Increase
10	27526	VALIUM10	CG8843	*Sec5*	*Secretory 5*	Increase
11	27530	VALIUM10	CG9012	***Chc***	***Clathrin heavy chain***	Increase
12	28941	GD	CG8725	*CSN4*	*COP9 signalosome subunit 4*	Increase
13	29316	VALIUM10	CG8053	*eIF1A*	*eukaryotic translation initiation factor 1 A*	Increase
14	29535	VALIUM10	CG3193	***crn***	***crooked neck***	Increase
15	29575	GD	CG1957	*Cpsf100*	*Cleavage and polyadenylation specificity factor 100*	Increase
16	29741	GD	CG33507	*dpr2*	*defective proboscis extension response 2*	Increase
17	30518	VALIUM10	CG3664	*Rab5*	*Rab5*	Increase
18	30600	GD	CG7471	*HDAC1*	*Histone deacetylase 1*	Increase
19	32854	VALIUM20	CG5374	*CCT1*	*Chaperonin containing TCP1 subunit 1*	Increase
20	33043	VALIUM20	CG9193	*PCNA*	*Proliferating cell nuclear antigen*	Increase
21	33615	VALIUM20	CG4006	*Akt1*	*Akt1*	Increase
22	34335	VALIUM20	CG3539	*Slh*	*SLY-1 homologous*	Increase
23	34356	VALIUM20	CG5706	*beta-PheRS*	*Phenylalanyl-tRNA synthetase, beta-subunit*	Increase
24	34567	VALIUM20	CG7885	*RpII33*	*RNA polymerase II 33kD subunit*	Increase
25	34582	VALIUM20	CG3762	*Vha68-2*	*Vacuolar H[+] ATPase 68 kDa subunit 2*	Increase
26	34710	VALIUM20	CG4579	*Nup154*	*Nucleoporin 154kD*	Increase
27	34711	GD	CG3806	*eIF2Bepsilon*	*eukaryotic translation initiation factor 2B subunit epsilon*	Increase
28	34730	VALIUM20	CG2051	*Hat1*	*Histone acetyltransferase 1*	Increase
29	34836	VALIUM20	CG4264	*Hsc70-4*	*Heat shock protein cognate 4*	Increase
30	34840	VALIUM20	CG16941	*Sf3a1*	*Splicing factor 3a subunit 1*	Increase
31	35804	VALIUM22	CG9191	*Klp61F*	*Kinesin-like protein at 61 F*	Increase
32	37609	GD	CG7583	*CtBP*	*C-terminal Binding Protein*	Increase
33	39335	GD	CG3193	***crn***	***crooked neck***	Increase
34	40691	GD	CG2038	*CSN7*	*COP9 signalosome subunit 7*	Increase
35	44288	GD	CG9033	*Tsp47F*	*Tetraspanin 47 F*	Increase
36	50565	GD	CG42522	*CSN8*	*COP9 signalosome subunit 8*	Increase
37	100315	KK	CG11092	*Nup93-1*	*Nucleoporin 93kD-1*	Increase
38	101404	KK	CG44436	*sno*	*strawberry notch*	Increase
39	101513	KK	CG3186	*eEF5*	*eukaryotic translation elongation factor 5*	Increase
40	103205	KK	CG1322	*zfh1*	*Zn finger homeodomain 1*	Increase
41	103383	KK	CG9012	***Chc***	***Clathrin heavy chain***	Increase
42	103767	KK	CG13387	*emb*	*embargoed*	Increase
43	104096	KK	CG14230	*CG14230*	—	Increase
44	105653	KK	CG2095	*Sec8*	*Secretory 8*	Increase
45	105763	KK	CG17737	*eIF1*	*eukaryotic translation initiation factor 1*	Increase
46	106144	KK	CG6094	*CG6094*	—	Increase
47	106240	KK	CG6382	*eRF3*	*eukaryotic translation release factor 3*	Increase
48	107264	KK	CG5081	*Syx7*	*Syntaxin 7*	Increase
49	109782	KK	CG10840	*eIF5B*	*eukaryotic translation initiation factor 5B*	Increase
50	110355	KK	CG7831	*ncd*	*non-claret disjunctional*	Increase
1	25950	VALIUM10	CG11556	*Rph*	*Rabphilin*	Decrease
2	25988	VALIUM10	CG2848	*Tnpo-SR*	*Transportin-Serine/Arginine rich*	Decrease
3	31893	VALIUM10	CG7178	*wupA*	*wings up A*	Decrease
4	32972	VALIUM20	CG13745	*FANCI*	*Fanconi anemia complementation group I*	Decrease
5	33662	VALIUM20	CG18174	*Rpn11*	*Regulatory particle non-ATPase 11*	Decrease
6	34727	GD	CG3889	*CSN1b*	*COP9 signalosome subunit 1 b*	Decrease
7	34788	VALIUM20	CG11985	*Sf3b5*	*Splicing factor 3 b subunit 5*	Decrease
8	35741	VALIUM20	CG5429	*Atg6*	*Autophagy-related 6*	Decrease

a Genes in bold font were identified more than once.

### Validation of RNAi line gene targets

The use of RNAi is a well-established experimental approach to quickly link genes with developmental functions, and our results with the reported lines are highly reproducible. However, it is possible that off-target effects of RNAi may be responsible for some of the observed phenotypes and may also account for differential RNAi effects between primary and secondary screens. A common genetic approach to validating RNAi phenotypes is to use additional lines targeting the same gene. Replication of the phenotype with multiple RNAi lines increases the likelihood of functional disruption of the target gene being the cause. While it was not feasible for us to do this type of cross-validation for the entire RNAi line collection, we attempted to validate a subset of lines in this manner. We obtained 14 new RNAi lines targeting genes that, when disrupted in mature blood cells (*Hml^Δ^-GAL4*) using screen RNAi lines, cause an increase in lymph gland *Hml^Δ^-DsRed* fluorescence. Subsequent analysis showed that 11 of 14 validation RNAi lines caused either the same or a highly similar phenotype as the original RNAi line (Supplementary Figure S1). Several such RNAi line cross-validations also appeared within the screen itself. For example, in the primary screen using *HHLT-GAL4*, seven genes (*zip*, *AP-2α*, *Rep*, *αSnap*, *Bap60*, *eIF3m*, and *CSN1b*) were identified by two different RNAi lines, and two genes (*Nup93-1* and *Chc*) by three different RNAi lines (Table 1). Secondary, cell-type-specific, screening also identified multiple RNAi lines targeting *CG14230*, *crn*, and *Chc* (Tables 2–4).

Additional evidence pointing to the validity of the RNAi lines identified in the screen is the independent identification of genes with linked functions. For example, the primary screen (*HHLT-GAL4*) identified several components of the COP9 Signalosome (CSN), an important negative regulator of cullin-RING ubiquitin ligases (Dubiel *et al.* 2020), namely *CSN1b* (identified twice), *CSN2* (*alien*), *CSN5*, and *CSN6* (Table 1). Likewise, the screen uncovered *Nup88* (*mbo*), *Nup93-1* (found twice), *Nup214*, and *Nup358*, genes encoding subunits (nucleoporins) of the Nuclear Pore Complex (NPC), which regulates nucleocytoplasmic shuttling, gene expression, and a variety of other cellular processes (Mondal *et al.* 2014; Kuhn and Capelson 2019; Cho and Hetzer 2020). Beyond multi-subunit complexes, many screen-identified genes delineated functional pathways or systems within the cell. For example, the collective identification of the genes *Clathrin heavy chain*, *shibire* (encoding Dynamin), *Amphiphysin*, three of four AP-2 adapter genes (*AP-2α*, *AP-2σ*, and *AP-2μ*), *Rab5*, and *Syntaxin7* suggests an important regulatory role for endosome formation and trafficking in hematopoiesis. The secondary screening, which examined the primary screen RNAi lines as well as additional RNAi lines targeting the same or related genes, identified *CSN5* and *CSN6* again (with *dome-GAL4*; Table 3), but also *CSN4*, *CSN7*, *CSN8*, and *Nup154* (with *Hml^Δ^-GAL4*; Table 4), among others. Thus, while RNAi off-target effects may account for some of the hematopoietic phenotypes we have observed, the results described above collectively point to the general validity of our RNAi line collection and reinforce our association of target gene function with hematopoiesis.

### Bioinformatic analysis of identified gene sets

To better understand the nature of the genes identified in the primary and the secondary screens, we analyzed each gene set using the online STRING protein interaction database (v11.0; Szklarczyk *et al.* 2019). Examination of the set of 137 genes identified in the primary screen using *HHLT-GAL4* revealed a significant enrichment of protein-protein interactions (PPIs) within this group (*P*-value < 1.0e–16; STRING 11.0). Not surprisingly, a large number of Gene Ontology (GO) terms, many of which are defined very broadly, were also found to be enriched within the Biological Process (456 GO terms), Molecular Function (40 GO terms), and Cellular Component (109 GO terms) categories (Supplementary Table S4). Comparison of our genes with the KEGG pathway database (Kanehisa and Goto 2000) offered a more refined view, identifying enrichment in eight defined functional pathways (Table 5), the most significant of which is RNA transport (KEGG dme03013; 13 of 139 genes, *q* = 1.35e–07). KEGG analysis also identified the Spliceosome (KEGG dme03040; 8 of 117 genes, *q* = 1.1e–04) and mRNA surveillance (KEGG dme03015; 6 of 72, *q* = 1.1e–03) groups, which, collectively, indicates an important role for RNA processing regulation during hematopoietic development. Gene set analysis by the Reactome pathway database (Fabregat *et al.* 2018), which defines almost three times the number of functional pathways as KEGG, identified 157 pathways to be enriched (Supplementary Table S5), 11 of which coincide with RNA regulation (Table 6). Another major functional theme uncovered by KEGG and Reactome analysis is vesicular trafficking. Three of the eight identified KEGG pathways were Endocytosis (KEGG dme04144; 9 of 119, *q* = 1.1e–04), Phagosome (KEGG dme04145; 7 of 83, *q* = 4.4e–04), and SNARE interaction in vesicular transport (KEGG dme04130; 3 of 20, *q* = 9.7e–03), while 14 related pathways were identified by Reactome (Table 6 and Supplementary Table S5).

**Table 5:** KEGG PATHWAY analysis^a^ of screen-identified hematopoietic gene sets

Driver Line	KEGG ID	Term description	Observed gene count	Background gene count	***q*-values** ^b^	Identified Genes	
***HHLT-GAL4 UAS-GFP***	dme03013	RNA transport	13	139	1.35E−07	*CG17737, CG3806, CG8636, Fs(2)Ket, Nup214, Nup358, Nup93-1, Ref1, eIF-1A, eIF2B-beta, eIF5B, emb, mbo*	
	dme04144	Endocytosis	9	119	0.00011	*AP-2alpha, AP-2mu, AP-2sigma, Amph, Chc, Hsc70-4, Rab35, Rab5, shi*	
	dme03040	Spliceosome	8	117	0.00044	*CG10333, CG11985, CG16941, CG6227, Hsc70-4, Prp19, Ref1, crn*	
	dme04145	Phagosome	7	83	0.00044	*CG7794, Rab5, Syx18, Syx7, Vha55, Vha68-2, mys*	
	dme03015	mRNA surveillance pathway	6	72	0.0011	*CG7185, Cpsf100, Elf, Ref1, Smg5, eRF1*	
	dme04213	Longevity regulating pathway—multiple species	5	54	0.0022	*Akt1, Hsc70-4, Rpd3, SNF4Agamma, chico*	
	dme04130	SNARE interactions in vesicular transport	3	20	0.0097	*Syx18, Syx1A, Syx7*	
	dme04120	Ubiquitin mediated proteolysis	5	99	0.0212	*Cdc27, Cul1, Prp19, shtd, slmb*	
***Antp-GAL4 Hml*** ^*Δ*^ ***- DsRed* *increase***	dme04144	Endocytosis	4	119	0.00012	*AP-2mu, Chc, Rab5, shi*	
	dme04145	Phagosome	2	83	0.0176	*Dhc64C, Rab5*	
	dme03013	RNA transport	2	139	0.031	*Nup358, Nup93-1*	
***Antp-GAL4 Hml*** ^*Δ*^ ***- DsRed* *decrease***	dme04330	Notch signaling pathway	2	22	0.0038	*CtBP, Rpd3*	
	dme04068	FoxO signaling pathway	2	65	0.0105	*babo, chico*	
	dme04213	Longevity regulating pathway—multiple species	2	54	0.0105	*Rpd3, chico*	
***dome-GAL4 Hml*** ^*Δ*^ ***- DsRed* *increase***	dme04145	Phagosome	3	83	0.0171	*Rab5, Syx7, Vha68-2*	
	dme04144	Endocytosis	3	119	0.0234	*AP-2mu, Chc, Rab5*	
***dome-GAL4 Hml*** ^Δ^ ***- DsRed decrease***	dme03040	Spliceosome	4	117	0.00013	*CG6227, Hsc70-4, Prp19, crn*	
	dme04120	Ubiquitin mediated proteolysis	2	99	0.0408	*Prp19, slmb*	
	dme04141	Protein processing in endoplasmic reticulum	2	130	0.0408	*Hsc70-4, Sec23*	
	dme04144	Endocytosis	2	119	0.0408	*Hsc70-4, shi*	
***Hml*** ^*Δ*^ ***-GAL4 Hml*** ^*Δ*^ ***- DsRed increase*** ^c^	dme03013	RNA transport	7	139	1.11E−05	*CG17737, CG3806, Nup154, Nup93-1, eIF-1A, eIF5B, emb*	
	dme04144	Endocytosis	6	119	4.03E−05	*AP-2alpha, AP-2sigma, Chc, Hsc70-4, Rab5, shi*	
	dme04213	Longevity regulating pathway—multiple species	3	54	0.0078	*Akt1, Hsc70-4, Rpd3*	
	dme04130	SNARE interactions in vesicular transport	2	20	0.0163	*Syx1A, Syx7*	
	dme04145	Phagosome	3	83	0.0163	*Rab5, Syx7, Vha68-2*	
	dme04330	Notch signaling pathway	2	22	0.0163	*CtBP, Rpd3*	
	dme03040	Spliceosome	3	117	0.0279	*CG16941, Hsc70-4, crn*	

a KEGG analysis via STRING v11.0.

b *q*-Values are false discovery rate-adjusted *P*-values.

c No KEGG groups were identified for the small decrease gene set for this GAL4 driver.

**Table 6:** Reactome analysis^a^ of *HHLT-GAL4* screen-identified hematopoietic genes

Functional Group	Reactome ID	Term description	Observed gene count	Background gene count	***q*-values** ^b^	Identified genes	
**RNA regulation**	DME-74160	Gene expression (transcription)	22	508	4.33E-07	*AGO1, Akt1, Bap60, CG7185, Cdk12, Cdk9, Cpsf100, Cul1, CycK, Dp, Nup214, Nup93-1, Prosalpha7, Rep, RpII33, Rpd3, Rpn11, Rpn6, SNF4Agamma, kni, mbo, msk*	
	DME-8953854	Metabolism of RNA	21	487	9.52E-07	*Akt1, CG10333, CG11920, CG11985, CG16941, CG6227, CG7185, Cpsf100, Elf, Hsc70-4, Nup214, Nup93-1, Prosalpha7, Prp19, RpII33, Rpn11, Rpn6, bys, crn, eRF1, mbo*	
	DME-72203	Processing of capped intron-containing pre-mRNA	13	218	1.35E-05	*CG10333, CG11985, CG16941, CG6227, CG7185, Cpsf100, Hsc70-4, Nup214, Nup93-1, Prp19, RpII33, crn, mbo*	
	DME-212436	Generic transcription pathway	15	343	5.57E-05	*Akt1, Bap60, Cdk12, Cdk9, Cul1, CycK, Dp, Prosalpha7, Rep, RpII33, Rpd3, Rpn11, Rpn6, SNF4Agamma, kni*	
	DME-73857	RNA Polymerase II Transcription	17	456	8.38E-05	*Akt1, Bap60, CG7185, Cdk12, Cdk9, Cpsf100, Cul1, CycK, Dp, Prosalpha7, Rep, RpII33, Rpd3, Rpn11, Rpn6, SNF4Agamma, kni*	
	DME-72163	mRNA Splicing—Major Pathway	10	169	0.00017	*CG10333, CG11985, CG16941, CG6227, CG7185, Cpsf100, Hsc70-4, Prp19, RpII33, crn*	
	DME-5578749	Transcriptional regulation by small RNAs	6	50	0.00023	*AGO1, Nup214, Nup93-1, RpII33, mbo, msk*	
	DME-450531	Regulation of mRNA stability by proteins that bind AU-rich elements	5	83	0.0067	*Akt1, Hsc70-4, Prosalpha7, Rpn11, Rpn6*	
	DME-191859	snRNP Assembly	3	31	0.0139	*Nup214, Nup93-1, mbo*	
	DME-72165	mRNA Splicing—Minor Pathway	3	46	0.0298	*CG10333, CG11985, RpII33*	
	DME-112382	Formation of RNA Pol II elongation complex	3	58	0.045	*Cdk9, CycK, RpII33*	
**Vesicular trafficking**	DME-199991	Membrane trafficking	25	359	8.76E-12	*AP-2alpha, AP-2mu, AP-2sigma, Akt1, Amph, CG7794, CSN1b, CSN5, CSN6, Chc, Hsc70-4, Rab35, Rab5, Rep, Sec23, Slh, Snmp1, Syx18, alien, alphaSnap, beta'COP, ctp, ldlCp, mgl, shi*	
	DME-8856828	Clathrin-mediated endocytosis	14	109	1.64E-09	*AP-2alpha, AP-2mu, AP-2sigma, Amph, CSN1b, CSN5, CSN6, Chc, Hsc70-4, Rab5, Snmp1, alien, mgl, shi*	
	DME-8856825	Cargo recognition for clathrin-mediated endocytosis	10	84	1.08E-06	*AP-2alpha, AP-2mu, AP-2sigma, CSN1b, CSN5, CSN6, Chc, Snmp1, alien, mgl*	
	DME-199977	ER to Golgi Anterograde Transport	7	86	0.00039	*CG7794, Sec23, Slh, alphaSnap, beta'COP, ctp, ldlCp*	
	DME-6798695	Neutrophil degranulation	14	408	0.00064	*Cdk12, EF2, Fs(2)Ket, Hsc70-4, Nup358, Rab5, Rpn11, Rpn6, Snmp1, TER94, Tsp47F, ctp, fabp, mys*	
	DME-983169	Class I MHC mediated antigen processing & presentation	10	216	0.00072	*Cdc27, Cul1, Prosalpha7, Rpn11, Rpn6, Sec23, Snmp1, mys, shtd, slmb*	
	DME-5620916	VxPx cargo-targeting to cilium	4	21	0.00085	*Exo84, Sec5, Sec6, Sec8*	
	DME-416993	Trafficking of GluR2-containing AMPA receptors	3	10	0.0018	*AP-2alpha, AP-2mu, AP-2sigma*	
	DME-6807878	COPI-mediated anterograde transport	5	61	0.0029	*CG7794, alphaSnap, beta'COP, ctp, ldlCp*	
	DME-8856688	Golgi-to-ER retrograde transport	5	65	0.0037	*CG7794, Syx18, alphaSnap, beta'COP, ctp*	
	DME-6811442	Intra-Golgi and retrograde Golgi-to-ER traffic	6	123	0.0067	*CG7794, Syx18, alphaSnap, beta'COP, ctp, ldlCp*	
	DME-6811434	COPI-dependent Golgi-to-ER retrograde traffic	3	29	0.0126	*Syx18, alphaSnap, beta'COP*	
	DME-432722	Golgi Associated Vesicle Biogenesis	3	31	0.0139	*Chc, Rab5, shi*	
	DME-204005	COPII-mediated vesicle transport	3	33	0.0152	*Sec23, Slh, alphaSnap*	

a Reactome analysis via STRING v11.0.

b *q*-Values are false discovery rate-adjusted *P*-values.

The numbers of candidate hematopoietic genes identified by the secondary screening, using cell-type-specific RNAi along with the *Hml^Δ^-DsRed* maturation marker, were fewer than the number of genes identified in the primary screening. However, when gene enrichment within the secondary screen rose to significance, it was very often in functional groups that were also enriched in the primary screen. Indeed, of the eight KEGG enrichment groups identified by the primary screening, all but one group (dme03015, *mRNA surveillance pathway*) were found to be enriched among the secondary screening gene subsets (Table 5). Three additional functional groups (dme04330, *Notch signaling pathway*; dme04068, *FoxO signaling pathway*; and dme04141, *Protein processing in endoplasmic reticulum*) were also found to be enriched specifically among the secondary screen gene subsets. *Notch signaling pathway* gene enrichment shows up twice, identified by RNAi knockdown in PSC cells by *Antp-GAL4*, and in maturing blood cells by *Hml^Δ^-GAL4* (Table 5). *FoxO signaling pathway* enrichment, like *Notch signaling pathway* enrichment, was identified by RNAi knockdown in PSC cells by *Antp-GAL4*, while enrichment for *Protein processing in endoplasmic reticulum* was identified by *dome-GAL4*-mediated RNAi in blood progenitor cells. With regard to phenotype, *Notch signaling pathway* gene enrichment identified by *Hml^Δ^-GAL4*-mediated RNAi was associated with an increase in *Hml^Δ^-DsRed* fluorescence, while the enrichment observed with *Antp-GAL4*-mediated RNAi was associated with a decrease in *Hml^Δ^-DsRed* fluorescence. As for *FoxO signaling pathway* (*Antp-GAL4*) and *Protein processing in endoplasmic reticulum* (*dome-GAL4*), both enrichments were associated with a decrease in *Hml^Δ^-DsRed* fluorescence. The *Hml^Δ^-DsRed* phenotypes associated with the seven functional enrichment groups overlapping with the primary screen (*HHLT-GAL4*) can be found in Table 5.

## Discussion

To find new genes regulating fly hematopoiesis, we have conducted a reverse-genetic screen using RNAi. The primary phase of our screen examined the role of approximately 3500 genes, representing about 25% of the genome. Functional gene disruption was achieved using the *GAL4/UAS* gene expression system, specifically the *HHLT-GAL4* driver, the activity of which is highly restricted to the lymph gland and circulating blood cell populations (Mondal *et al.* 2014). In any experimental context, direct examination of affected tissues is best, however the dissection of lymph glands for this purpose is relatively difficult and time consuming, especially for undergraduates without prior experience. Thus, we elected to circumvent dissections by taking advantage of the translucent nature of larvae and screening whole animals for GFP expression in the cells of the hematopoietic system (via *HHLT-GAL4 UAS-GFP*). While indirect, this approach was advantageous because general lymph gland morphologies and circulating cell densities could be easily evaluated and compared across genotypes *in situ*, while also increasing the analytical throughput (Figure 2). Ultimately, this screen identified 137 candidate genes, corresponding to 148 different RNAi lines, which broadly regulate hematopoietic development (Table 1).

With the 148 RNAi lines identified in the primary screen, we set out to refine our understanding of where each candidate gene was functioning in the lymph gland (i.e., whether in PSC cells, the progenitor cells, or the mature cells), and of how its functional disruption impacted lymph gland development. To achieve this, we first added additional RNAi lines (54; Supplementary Table S1) that were either redundant or targeted genes that were functionally related to candidate hits from our primary screen. Second, we generated new *GAL4* driver lines that (1) target RNAi to lymph gland sub-populations and (2) report on the development of mature blood cells. Our collection of 202 RNAi lines was then screened using these driver lines (*Antp-GAL4*, *dome-GAL4*, and *Hml^Δ^-GAL4*, each with *Hml^Δ^-DsRed* in the background), and DsRed fluorescence was evaluated in whole animals, similar to GFP in the primary screen (see Figures 3–5). Each *GAL4* driver identified target gene subsets that, when disrupted in their respective cell types, increase or decrease in DsRed fluorescence (Tables 2–4), changes that typically appeared to correlate with lymph gland size. However, for RNAi backgrounds with reduced fluorescence, we cannot rule out the possibility that lymph glands were normal in size or even enlarged but exhibited reduced *Hml^Δ^-DsRed* expression.

Previous work by several groups has demonstrated that the PSC communicates with both lymph gland progenitor cells and differentiating/mature cells to regulate development (Krzemień *et al.* 2007; Mandal *et al.* 2007; Mondal *et al.* 2011; Benmimoun *et al.* 2012; Pennetier *et al.* 2012; Tokusumi *et al.* 2012), and our findings here are consistent with this role. Reduction of gene function in PSC cells (*Antp-GAL4*) identified 34 genes regulating blood cell maturation and/or proliferation in the lymph gland, 19 causing an increase and 15 causing a decrease in *Hml^Δ^-DsRed* expression (Table 2). Since PSC cells support blood development but never contribute to the blood cell pool (Jung *et al.* 2005), each of the genes identified presumably plays a direct or a indirect role in signaling mechanisms regulating hematopoiesis. Perhaps not surprisingly, RNAi directly in lymph gland blood progenitor cells (*dome-GAL4, dome^MESO^-GAL4*) also identified a number of candidate genes regulating blood cell maturation. Specifically, progenitor-cell RNAi identified 50 genes, 33 that cause an increase and 17 that cause a decrease in *Hml^Δ^-DsRed* fluorescence in the lymph gland. Previous work has shown lymph gland progenitor cells to be regulated by several paracrine and metabolic signaling mechanisms (Owusu-Ansah and Banerjee 2009; Sinenko *et al.* 2009, 2011; Mondal *et al.* 2011; Tiwari *et al.* 2020), so it will be interesting to address potential connections to our candidate genes in future work. Targeting gene knock down to differentiating and mature blood cells (*Hml^Δ^-GAL4*) identified the largest cell-type-specific subset with 56 candidate genes, 48 of which increase and 8 of which decrease *Hml^Δ^-DsRed* fluorescence in the lymph gland. Previous work has demonstrated that interaction between mature cells and progenitor cells, via the “equilibrium signaling pathway,” is important for balancing progenitor cell maintenance and differentiation (Mondal *et al.* 2011, 2014). Blocking equilibrium signaling in maturing cells leads to a compensatory proliferation and differentiation of progenitor cells (Mondal *et al.* 2011, 2014). Thus, the increase in *Hml^Δ^-DsRed* fluorescence in whole animals, upon functional disruption of genes in the mature blood cell population, suggests that many of them may play a role in equilibrium signaling. Although we cannot be certain of the specific roles of the identified genes in each cell population, our dataset provides a valuable starting point for asking these questions.

It is interesting to note that many of the RNAi lines identified in the primary screen using *HHLT-GAL4* were not identified (did not cause a phenotype) by any single *GAL4* driver in the secondary, cell type-specific screen. One possible reason is that *HHLT-GAL4* phenotypes for most RNAi lines are complex, arising only because of functional disruption in multiple cell types simultaneously. Another possibility is a threshold effect owing to differences in *GAL4* driver strength, i.e., the individual cell-type *GAL4* drivers may not induce RNAi as robustly as *HHLT-GAL4*. For RNAi lines that do cause phenotypes both with *HHLT-GAL4* and with a cell type-specific *GAL4* driver, it is not clear that these are equivalent phenotypes. The absence of a hematopoietic marker in the *HHLT-GAL4* screen and the differences in GAL4 expression levels and patterns contribute to this uncertainty. Thus, while we are confident that the candidate hematopoietic genes identified by *HHLT-GAL4* in the primary phase of the screen are valid, it seems that determining the functional specificities of candidate genes may be more straightforward for those causing phenotypes when disrupted in a single hematopoietic cell type.

Our analysis of the primary screen candidate genes using the online STRING database helped to reveal important genes subsets. The protein–protein interaction (PPI) network for our 137 gene dataset is composed of 599 edges (known or predicted interactions), a number significantly greater than the 350 edges expected for a randomly selected network of the same size (*P*-value = 1.0e–16). Likewise, large numbers of Gene Ontology terms were also enriched for this network (Supplementary Table S4), though many of the terms are broad and overlapping. However, network analysis using the KEGG Pathway database identified a smaller number of enriched functional groups or pathways. Of the eight groups identified by KEGG analysis (Table 5), three pointed to mRNA maturation (*RNA transport*, KEGG dme03013; *Spliceosome*, KEGG dme03040; and *mRNA surveillance*, KEGG dme03015) and another three pointed to vesicular trafficking (*Endocytosis*, KEGG dme04144; *Phagosome*, KEGG dme04145; and *SNARE interaction in vesicular transport*, KEGG dme04130) as having major hematopoietic roles.

Despite smaller gene sets from the secondary screening, seven of the eight primary screen KEGG enrichment pathways were identified again in these genes (Table 5), underscoring the relevance of these functional groups. KEGG analysis of the secondary screen candidate gene subsets also identified three additional enriched functional groups, *Notch signaling pathway* (dme04330), *FoxO signaling pathway* (dme04068), and *Protein processing in endoplasmic reticulum* (dme04141). It is interesting that *Notch signaling pathway* was identified twice by RNAi, once in the PSC (*Antp-GAL4*) and once in maturing cells (*Hml^Δ^-GAL4*), as both cell types have known roles for Notch signaling during hematopoiesis (Lebestky *et al.* 2003; Mukherjee *et al.* 2011; Ferguson and Martinez-Agosto 2014; Blanco-Obregon *et al.* 2017). Finding enrichment of *FoxO signaling pathway* by RNAi in the PSC (*Antp-GAL4*) is based upon identifying the genes *chico*, encoding the Insulin Receptor Substrate homolog, and *babo*, encoding the TGF-β/Activin receptor (Table 5). Insulin signaling has been shown to regulate both lymph gland progenitor cell and PSC cell populations (Benmimoun *et al.* 2012; Shim *et al.* 2012; Tokusumi *et al.* 2012; Kaur *et al.* 2019), though Chico function itself has not been previously analyzed. While the evidence for TGF-β/Activin signaling is lacking, the PSC population is known to be regulated by TGF-β/Dpp signaling (Pennetier *et al.* 2012). Others have shown that the gene *dawdle*, encoding an Activin-like ligand that activates Babo, is directly regulated by FoxO (Bai *et al.* 2013), raising the possibility that the Insulin and TGF-β/Activin pathways converge in PSC cells.

Our screening and bioinformatic analyses have identified candidate hematopoietic genes but have also brought to light what appear to be broader realms of hematopoietic regulatory control. We have found that the areas of endosomal trafficking, mRNA regulation, and the ubiquitin-ligase system each have a number of constituent genes that control blood cell development in some way, including a smaller number of genes that are uniquely positioned at functional interfaces between these larger realms. The case for endosomal trafficking was made previously, in part, in the discussion of our gene set validation; however a number of other genes belonging to this group were not mentioned, including those encoding a variety of other Rab and Rab effector proteins, syntaxins (SNAREs), and a multifunctional chaperone called Hsc70-4. It is well established that functional disruption of early endosomal trafficking (e.g., mutation of *Syx7* or *Rab5*) can cause a variety of cellular defects including loss of apicobasal polarity, increased proliferation, and aberrant activation of signaling pathways such as Notch and EGFR (Vieira *et al.* 1996; Lu and Bilder 2005; Vaccari and Bilder 2005; Fortini and Bilder 2009; Reimels and Pfleger 2015). The finding of *Hsc70-4* stands out because it is a known regulator of Notch signaling (Hing *et al.* 1999), important in hematopoiesis (Duvic *et al.* 2002; Lebestky *et al.* 2003; Mandal *et al.* 2004; Mukherjee *et al.* 2011; Ferguson and Martinez-Agosto 2014; Small *et al.* 2014; Blanco-Obregon *et al.* 2017), but has also been functionally linked to clathrin-mediated vesicle formation and mRNA splicing (Chang *et al.* 2002; Herold *et al.* 2009).

Our screen identified an abundance of mRNA regulatory proteins involved in splicing, transport, translation initiation, and translation termination (Tables 5 and 6). The genes *crn* (the *Drosophila* homolog of the yeast *Clf1p* splicing factor) and *Prp19* are interesting because both encode components of the NineTeen Complex (NTC; Chanarat and Sträßer 2013), a key mRNA splicing regulator, and both are bridges to the ubiquitin-ligase system. In *Drosophila*, Crn is positively regulated by the HIB-Cul3 E3 ubiquitin ligase downstream of Hedgehog signaling (Liu *et al.* 2014), a key pathway controlling lymph gland hematopoiesis (Mandal *et al.* 2007). Prp19 itself is an E3 ubiquitin ligase, the activity of which is required for the proper assembly and activation of the spliceosome (Chan 2003; de Moura *et al.* 2018). While function of Prp19 in lymph gland hematopoiesis remains unclear, Prp19 has been shown to be required for proper Ras/MAP kinase signaling in the *Drosophila* eye, and for proper Notch signaling in the *C. elegans* germline (Ashton-Beaucage *et al.* 2014; Gutnik *et al.* 2018). Furthermore, mutation of *Prp19* was previously shown to cause a reduction in the crystal cell lineage during head mesoderm hematopoiesis in *Drosophila* embryos (Milchanowski *et al.* 2004). Several other ubiquitin-ligase system component genes were identified in our screen, including *Cdc27* and *shattered* (*shtd*; both part of the Anaphase Promoting Complex/Cyclosome E3 ubiquitin ligase), as well as *supernumary limbs* (*slmb*; encoding an F-box protein) and *Cullin 1* [*Cul1*; both part of the Skp/Cullin/F-box (SCF) subfamily of cullin-ring E3 ubiquitin ligases] (Petroski and Deshaies 2005). As mentioned previously, the CSN complex is a major regulator of the ubiquitin-ligase system (Petroski and Deshaies 2005; Dubiel *et al.* 2020), and our screen identified seven of nine *CSN* genes. In further support of a hematopoietic function for these genes, *Prp19*, the SCF E3 ubiquitin ligase components *SkpC* and *Cul4*, and *CSN1b* were previously identified in a screen for *Drosophila* melanotic tumor suppressor genes (Avet-Rochex *et al.* 2010).

Nucleoporins have been shown to mediate many important functions, including the production, transport, and translation of mRNAs (Kuhn and Capelson 2019; Cho and Hetzer 2020). In the context of *Drosophila* hematopoiesis specifically, the nucleoporin Nup98 has been shown to regulate Pvr expression, the receptor tyrosine kinase controlling equilibrium signaling in the lymph gland (Mondal *et al.* 2014). In humans, the normal hematopoietic roles of nucleoporins remains elusive, however several chromosomal translocations into nucleoporin genes, *Nup98* in particular, are known to cause a variety of hematopoietic defects and leukemias (Gough *et al.* 2011; Takeda and Yaseen 2014). Thus, the identification of several different nucleoporins in our screen confirms and extends the finding that these are important regulatory genes in the context of blood cell development.

The secondary phase of our screen began the work of identifying the specific cell types in which these genes function, as well as indicating whether the genes normally promote or limit the blood cell maturation process. Our findings also indicate that many of these candidate hematopoietic genes also control cellular proliferation, as lymph gland size and circulating cell densities were often changed. In the future, it will be important to examine these RNAi phenotypes again with additional hematopoietic markers, as many are likely to impact the differentiation of the crystal cell and lamellocyte lineages. For phenotypes with enlarged lymph glands with strong increases in *Hml^Δ^-DsRed* expression, our experience suggests that progenitor cells are likely reduced or perhaps even missing. Thus, it will also be important in future analyses to test this hypothesis by using a progenitor cell marker, such as *dome^MESO^-GFP*, to directly assess these RNAi phenotypes. Characterization of the RNAi phenotypes described here will also benefit significantly from direct observation of lymph glands through dissection and higher-magnification microscopy. This is critical because the presence of small cell populations in the lymph gland, for example, remnant progenitor cells expressing *dome^MESO^-GFP*, have correspondingly low fluorescence levels and are impossible to see in whole-animal analyses. Dissection analysis will also provide insight into lymph gland structural changes and abnormal morphologies that arise in these RNAi phenotypes.

The genetic screen reported here was conducted by the UCLA Undergraduate Research Consortium for Functional Genomics (URCFG; Chen *et al.* 2005), which consists of students participating in *Biomedical Research 10H*, a course-based undergraduate research experience (CURE) offered by the UCLA Minor in Biomedical Research. This RNAi-based screen for new hematopoietic genes represents the third iteration of a CURE-based pedagogical approach to teaching UCLA URCFG undergraduates about science and scientific research. The two previous research projects completed by the URCFG were mosaic analysis of lethal P-element insertional mutants in the fly eye (Chen *et al.* 2005; Call *et al.* 2007) and *in vivo* cell lineage tracing during *Drosophila* development using G-TRACE (Evans *et al.* 2009; Olson *et al.* 2019).

As an educational tool, this screen featured several design aspects that made its implementation as a CURE research project possible. CUREs strive to provide an authentic research experience for undergraduates, but this can be difficult to achieve if students work as research apprentices cultivating individual projects. We have found that research authenticity is much more manageable when students work in parallel, performing the same kind of experimental work, but collecting unique data, and that genetic screens reflect this approach well. The use of RNA interference (RNAi) as the basis for the genetic screen was particularly beneficial. Using RNAi in the context of the *GAL4/UAS* system enabled students to conduct an F1 screen, allowing for more throughput within the UCLA 10-week academic quarter. It also allowed us to take advantage of the thousands of transgenic GAL4-responsive RNAi fly lines that were already available to the fly research community. RNAi-based screening also provided students with a direct link to target gene identities and known functions. While screening was ongoing, students learned how to identify target genes associated with their RNAi fly stocks, how to mine FlyBase for information about their target genes, and how to use NCBI BLAST to identify human homologs. Lastly, the selection and the use of the highly specific *HHLT-GAL4 UAS-GFP* and *Hml^Δ^-DsRed* reporter lines was advantageous, as it allowed students to screen for hematopoietic phenotypes directly in translucent larvae, bypassing difficult and time-consuming dissection and tissue processing procedures.

**Figure 6:**
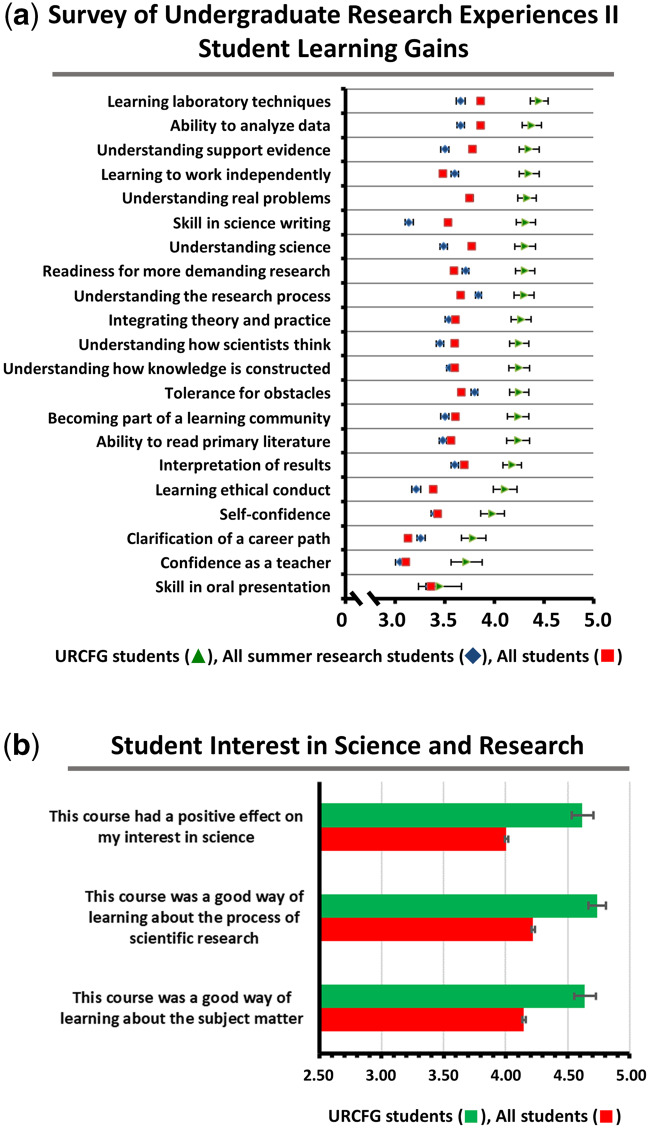
Impact of the URCFG experience on learning gains. (A) Categorical data plot comparing reported learning gains between URCFG students (green triangles), students, nationally, completing summer research apprenticeships (all summer research students; blue diamonds), and students, nationally, completing introductory to advanced biology courses containing some research component (all students; red squares). Students participating in the URCFG who responded to the survey (*n* = 265) reported increased gains across 20 of 21 different areas compared to students in the other groups. Scale: 1 = little to no gain, 2 = small gain, 3 = moderate gain, 4 = large gain, and 5 = very large gain. Error bars represent two times the standard error, representing greater than a 95% confidence interval. (B) average responses of URCFG students (green bars, top), when asked if they agreed with each of the statements on the left, regarding the impact of the course on their interest in science, ability to learn the process of scientific research and ability to learn the subject matter. Students scored each statement on a 5-point Likert scale, where 1 is “strongly disagree” and 5 is “strongly agree.” Scores are compared to those from students nationally in biology courses with a research component (red bars, bottom). See *Materials and Methods* for additional details.

To explore how students might benefit from participating in the RNAi screen, we used the SURE II survey (Lopatto 2004), which assesses learning across 21 different areas for students participating in undergraduate research pedagogies. We find that URCFG students participating in our RNAi screen for hematopoietic genes reported increased learning gains in almost every area (20/21, as compared to national benchmarks; Figure 6A), a finding that is similar to the increased learning gains reported by undergraduates participating in our previous URCFG research pedagogies (Chen *et al.* 2005; Call *et al.* 2007; Olson *et al.* 2019). It is also noteworthy that URCFG students who participated in this project reported relative increases in their interest in science and scientific research (Figure 6B).

An increasingly important measure of the effectiveness of science pedagogies, including CUREs, is the impact that these pedagogies have on the retention of students in science, technology, engineering, and mathematics (STEM) majors. It has been previously reported that the STEM retention rate nationally (through degree completion) is approximately 40%, dropping to as low as 25% among underrepresented minority (URM) students (Hurtado *et al.* 2009; National Academies 2011; PCAST 2012). As recently reported (Olson *et al.* 2019), student participation in a URCFG CURE experience, including the one described here, correlates with an overall persistence of students in STEM majors at a rate that is greater than twice the national average (to 95%, *n* = 626). For URM students in particular, the increase in STEM retention is even greater (to 91%, *n* = 46). Our findings add to a growing body of evidence that authentic research experiences in the classroom context create highly effective learning environments for undergraduate students and can improve engagement and persistence in STEM (Chen *et al.* 2005; Call *et al.* 2007; Lopatto *et al.* 2008; Graham *et al.* 2013; Jordan *et al.* 2014; Shaffer *et al.* 2014; Rodenbusch *et al.* 2016; Olson *et al.* 2019).

## Notes

Student contributions: primary *HHLT-GAL4* screen, A. Abbasi—Z. Zhou; secondary cell-type-specific *GAL4* screen, M. Abutouk—J. Zimmerman; Summer and high school scholars, C. Bracken—D. Yim.
